# Toilet chemical additives and their effect on faecal sludge characteristics

**DOI:** 10.1016/j.heliyon.2020.e04998

**Published:** 2020-09-23

**Authors:** Eugene Appiah–Effah, Godwin Armstrong Duku, Bismark Dwumfour–Asare, Isaac Manu, Kwabena Biritwum Nyarko

**Affiliations:** a Regional Water and Environmental Sanitation Centre, Department of Civil Engineering, Kwame Nkrumah University of Science and Technology, Kumasi Ghana; b Department of Civil Engineering, Kwame Nkrumah University of Science and Technology, Kumasi Ghana; c Department of Environmental Health and Sanitation Education, University of Education Winneba, Asante–Mampong Campus Ghana

**Keywords:** Environmental engineering, Environmental health, Environmental management, Environmental pollution, Microbiology, Public health, Calcium carbide, Lambda-cyhalothrin, Faecal sludge, Toilet additive, Characterisation, Mass loss, Sanitisation, Public toilet, Biodegradation, Helminth eggs

## Abstract

This study investigated the effects of two mostly improvised chemical additives, calcium carbide and lambda super 2.5 EC (LSEC), on the physico–chemical and microbial characteristics of faecal sludge from toilets. The quality of faecal sludge was assessed before and after application of the chemical additives in an experimental setup of ten different treatment units including a control, and treatment replicates. The initial characteristic of the faecal sludge was slightly acidic with high content of slowly degradable organic matter. The experimental control without additives after 30 days showed reduction in BOD_5_, COD, helminth eggs and sludge mass by a maximum of 30%, 34.7%, 99.8% and 55% respectively. Similarly, calcium carbide additive reduced the BOD_5_, COD, helminth eggs and the mass of the faecal sludge by 47.4%, 48.3%, 99.6% and 61% respectively. Also, LSEC additive reduced BOD_5_, COD, helminth eggs and the mass of the sludge by 40.6%, 47.9%, 95.9% and 58% respectively. The two additives showed significant treatment effect on the faecal sludge although the level of treatment could not meet the regulatory discharge limits for the key quality parameters assessed including sanitisation. The study is still a grey area and more research is recommended to enrich the findings.

## 1 Introduction

In recent years, the use of toilet additives to treat faecal sludge in domestic sanitation facilities has received increasing attention [1, 2, 3] partly due to large sludge generation and stringent environmental management regulations [4]. Domestic faecal sludge is raw or partially digested slurry or semisolid usually generated from on–site toilet systems [5]. Major faecal sludge problems include finding safe handling and appropriate disposal options especially in developing countries [2]. Untreated faecal sludge is laden with pathogens which could cause illnesses such as diarrhoea, cholera and dysentery [1]. Safe disposal of faecal sludge into the environment will require treatments to sanitise (kill pathogens) and also to stabilise faecal matter (reduce vector attraction) [1, 6].

According Grolle et al. [7], toilet additives whether in their inorganic or organic forms have found application in enhancing faecal sludge treatment. These additives increase processes like faecal sludge decomposition and dewatering which result in reduction of sludge volume and pathogenic loads [1, 8, 9]. Chemical and biological processes involving the use of additives like ash, urea, lime, and lactic acid have been investigated for their efficacy in treating faecal sludge [6, 8, 9, 10]. Some studies have concluded that the additives ash, urea, lime and lactic acid can sanitise faecal sludge safely [3, 9, 11, 12]. Meanwhile, wide range of other additives are on the market with claims of capabilities for sludge stabilisation and sanitisation enhancement [13]. In addition to such treatment attributes are abilities to reduce odour and repel flies through the actions and processes of enzymes, microorganisms, and chemical reactions [13, 14]. Despite these promising claims, the effectiveness of toilet additives in reducing sludge volume is still under rigorous interrogation and debates [3]. Some studies on additives capabilities give mixed results - while some link significant sludge volume reduction and sanitization to additives [6, 15, 16], others claim otherwise or at best state “no evidence linking additives to faecal sludge decomposition and sanitization [1, 2, 7, 17, 18]. Kemboi et al., [1] however, attribute the differences in results to the difference in the active compounds/enzymes found in additives and the methods of application, yet that is a valid contribution to the ongoing intellectual debate.

Ghana like other developing countries should be interested in the ongoing debate on faecal sludge additives application. Knowledge from successful stories could be used to tackle some of the sanitation challenges that are particularly found in low–income urban areas [3, 18]. Majority of Ghanaians (67%) use shared toilet facilities including public and shared compound toilets [19, 20], and these have faster sludge accumulation and desludging frequencies with accompanied high cost burdens and poor toilet conditions [3, 21, 22, 23]. Meanwhile, limited studies exist on use of additives in faecal sludge treatment in Ghana. A study by Awere and Edu-Buandoh [3] suggested that additives are not effective treatment enhancers especially in faecal sludge degradation. Yet, some Ghanaians believe that the use of additives and such products once suggested by artisans and/or neighbours might be beneficial.

In Kumasi, the second largest city in Ghana, curiosity out of personal observation and communication with some peri–urban households revealed that some households improvise the use of calcium carbide and Lambda Super 2.5 EC (an agrochemical containing the active compound lambda–cyhalothrin) as additives to treat faecal sludge and excreta. According to them, some artisans and/or business enterprises involved in toilet construction prescribe these chemicals to their customers with claims that they are effective in reducing sludge volume in toilets. Meanwhile, there is no empirical study on the efficacy of the application of these chemicals as additives for faecal sludge or excreta treatment. This paper therefore presents a bench study that seeks to establish evidence of the efficacy of the two chemical agents currently improvised as faecal sludge additives.

## 2 Materials and methods

### 2.1 Faecal sludge sources and sample collection

Samples of faecal sludge (FS) were taken from the only three (3) functional public toilets at the Ayeduase and Kotei communities, which are all suburbs of the Oforikrom Municipal Assembly in Kumasi, Ghana. The two communities are neighbours to the Kwame Nkrumah University of Science and Technology (KNUST), the institution where the experimentation was done. Ayeduase and Kotei are known as dormitory towns because of their high student population. The majority (70%) of the inhabitants are served by on–site sanitation systems such as public toilets with technologies like Kumasi ventilated improved pits (KVIPs), and pour–flush and cistern flush toilets connected to septic tanks. One of the key challenges faced by managers of these public sanitation facilities is cost burden associated with the management of faecal sludge generated. The toilet facilities receive high usage rates [24], with quickly filled up pits and always requiring frequent desludging incurring higher cost [3, 22, 23, 25]. The challenging situation was same for the only functional toilets from which faecal sludge samples were taken at the time of the study. The three (3) toilets were desludged within short periods of every 2–3 weeks.

Samples of fresh faecal sludge were taken from the pits of public toilets at about one metre beneath the pit's pedestal in the morning between 7:30 and 8:00 am. This was immediately after the peak visiting hours when majority of users were expected to have finished using the facilities. In collecting the sludge samples, a five–point sampling was implored by arbitrarily spreading out the sampling points to five (5) different locations in each pit of the public latrines. Samples collected were stored in air–tight sterile plastic containers which were already rinsed thoroughly with distilled water. Adequate samples were collected from all three sources (6kg per toilet) and then thoroughly mixed by stirring to obtain a homogenous composite sludge sample. The pH and temperature values of the homogenous composite samples were then measured in–situ with the help of a handheld multi–parameter test kit [26]. The composite sludge was securely transported under storage condition of 4 °C to the Environmental Quality Laboratory of KNUST, where the content of the sludge was analysed for physico–chemical parameters including moisture content (MC), biochemical oxygen demand (BOD_5_), and chemical oxygen demand (COD); and microbial constituents like total coliforms (TC) and helminth eggs (HE), all within 24 h [27].

### 2.2 Experimental setup and laboratory analyses

The experimental setup and laboratory analyses followed standard procedure described by Foxon et al., [17]. The main additives tested in this study were calcium carbide and lambda super 2.5 EC (LSEC). The two chemicals were obtained from the open market in Kumasi. The calcium carbide was obtained in powdered form as commonly sold in the market. The LSEC was obtained from an agrochemical shop, and the main composition was read as lambda cyhalothrin, surfactants, creslox AE 4 (a mixture of calcium salt of alkyl benzene sulphonate, alkyl benzene ethoxylate and solvents), methanol isobutanol and aromex according to the manufacturer [28].

The experiment setup contained thirty (30) glass jars (with diameter of 10 cm and depth of 16.5 cm) consisting of triplicates of the nine (9) treatments (making 27) and a triplicate of the control (Table 1). Different dosages of the additives were prepared and separately applied to 300 g of faecal sludge samples in the glass jars. Details of the treatments and dosages are presented in Table 1. For the LSEC additives, 6 different treatments were prepared and investigated. In the first three treatments 25 mL, 50 mL and 75 mL volumes of the stock LSEC solution were applied separately to 300 g of faecal sludge in different glass jars. For the remaining three LSEC treatments, 50 mL of diluted LSEC with dilution factors (DF) 0.1%, 0.5% and 1% were separately applied to 300 g of faecal sludge in different glass jars. The diluted LSEC solutions were prepared by adding 1 mL, 5 mL and 10 mL of the stock LSEC to 1 L of distilled water to obtain 0.1%, 0.5% and 1% dilution factors respectively.

**Table 1:** Treatments description.

Chemical additive type & control	Treatments	Description of treatments
Stock LSEC	L1	25 mL of stock LSEC in FS
	L2	50 mL of stock LSEC in FS
	L3	75 mL of stock LSEC in FS
Diluted LSEC	LW1	50 mL of diluted LSEC (of dilution factor, 0.1%)
	LW2	50 mL of diluted LSEC (of dilution factor, 0.5%)
	LW3	50 mL of diluted LSEC (of dilution factors, 1.0%)
Calcium Carbide	C1	5 g of calcium carbide in FS
	C2	8 g of calcium carbide in FS
	C3	10 g of calcium carbide in FS
Control	CN	Control (faecal sludge without any additive)

For the calcium carbide additive, crystalline powder of quantities 5 g, 8 g and 10 g were separately applied to 300 g of faecal sludge in separate glass jars. The control was glass jars containing 300 g of faecal sludge without any additives. All the setups including the control were exposed to the same experimental conditions. The study was carried out under aerobic conditions (i.e. the surfaces of the glass jar setups were fully exposed/opened to air) under ambient air temperature [2, 17]. The setups were left in the experimental room and monitored for physico–chemical including mass loss, and microbial parameters for 30 days. For mass loss analyses, the mass or weight of the jars was recorded weekly [14] over a 4 week period. The rate of mass loss was calculated as the change in mass of jar content over the defined periods for each jar [2].

### 2.3 Characterisation of raw and treated faecal sludge

Raw and treated faecal sludge was characterised before and after application of the chemical additives. The physio–chemical and microbial parameters considered were mass loss, temperature, pH, biological oxygen demand (BOD_5_), chemical oxygen demand (COD), moisture content (MC), total coliforms (TC) and helminth eggs (HE). All parameters were measured in the laboratory using standard methods [27]. The pH and temperature of the raw sludge were measured with a multi–parameter pH meter (HI98196) every other day for fifteen (15) days. The rest of the parameters (MC, BOD_5_, COD, TC, HE and mass loss) were measured weekly for 4 weeks. Moisture content was determined by drying samples in an oven at 105 °C for about 24 h. The COD and BOD_5_ analyses were done using the closed reflux titrimetric and dilution methods respectively [29]. Helminth eggs were enumerated using a combination of the floatation and sedimentation method. Identification of helminths eggs was done using shape and size from bench aids for Diagnosis of Intestinal Parasites [30].

### 2.4 Data analysis

Microsoft Excel was used to process and analyse the data. Descriptive statistics in tables and graphs were used to describe the results. Also, inferential statistical tool such as Multivariate analysis of variance (MANOVA) from SPSS IBM Mac version 21 was used to identify any significant differences among treatments. The MANOVA test was used to analyse the key faecal sludge characteristics of moisture content (MC), biochemical and chemical oxygen demands (BOD_5_ & COD), total coliforms (TC), and helminth eggs (HE) to ascertain the extent of treatment achieved due to the additive applications. There were also Tests of Between-Subjects Effects and Multiple Comparisons test using the Least Significant Difference (LSD) [31]. Significant testing was done at 5% significance level. All the statistical tests generated especially for the five key sludge characteristics are shared as Supplementary material to the paper (see supplementary Tables A1–A4).

### 2.5 Limitation of the study

The chemical composition of the two additives used in the study was not verified especially for content in BOD_5_ and COD. The assumption was that the control (without additives) could offer explanations to differences due to the additives and otherwise. Faecal sludge from the only three available and functional public toilets in the study area at the time of the study provided limited scope and sample size. The study was carried out in a short period of 30 days close to existing desludging period of 2–3 weeks. Proof of additives’ efficacy within desludging period may be useful and justify their application to avert the cost burden of frequent public toilet desludging. The findings cannot be extrapolated beyond the study period. Furthermore, the experimental setup using glass jars may not be exact representations of real–world toilet pits, however, it could provide at least both the aerobic and anaerobic conditions.

## 3 Results and discussions

### 3.1 Characteristics of raw faecal sludge

The characteristics of the raw faecal sludge sample is presented in Table 2. The mean temperature value measured 25 °C corresponded to the ambient temperature. The raw sludge was slightly acidic (pH 6.5–6.6) with a narrow range. This pH range supports the claim that pH of sludge could be slightly acidic and/or near alkaline [26, 32, 33]. However, Appiah–Effah et al., [26] indicated that a lower pH value for public toilet sludge could result from acidic detergents used for toilet cleaning. Interaction with the toilet caretakers during the sampling revealed that toilets were cleaned with detergents prior to sampling like every other morning. Thus, likely contributing to the acidic nature of the sampled sludge in this study. Mean sludge moisture content was 85.94 ± 2.13% and the finding corroborate earlier studies that MC of fresh faecal sludge could be within 50–90% [3, 5, 18, 34]. A high moisture content according to Bakare et al [35] and Nabateesa et al [36] provides a suitable environment for microbial activity which could be useful for sludge decomposition. The high BOD_5_ and COD contents around 53,000 mg/L and 188,000 mg/L respectively were consistent with other studies on faecal sludge in Kumasi [26, 32, 37]. The BOD_5_ and COD values could be partly because the sludge was fresh and largely undigested. This is possible because it was confirmed during sampling that the public toilets were desludged between 1 and 3 weeks, therefore organic loads could only be partially degraded or not [25]. Meanwhile, the biodegradability ratio (BOD_5_/COD) ratio was around 0.3, a low value indicating that the sludge is largely composed of slowly degradable substances [29]. Thus confirming the assertion that human faeces contain slowly biodegradable organic matter as COD [35, 38, 39]. The implication is that microbial decomposition may be slower especially when given a short reaction period. The average total coliforms and helminth eggs in the raw sludge were measured as 128 × 10^6^ cfu/100 mL and 458 egg/gTS respectively. These microbial concentrations undoubtedly high especially the helminth eggs considering the stricter environmental discharge limit of <1 eggs/gTS given by Ghana's Environmental Protection Agency (EPA–GH).

**Table 2:** Characteristics of raw faecal sludge.

Parameters	Mean	Minimum	Maximum	Standard deviation
Temperature (^o^C)	25	25	25	-
pH	6.5	6.5	6.6	-
MC (%)	85.94	84.63	88.40	2.13
BOD_5_ (mg/l)	52,668.7	46,410	56,836	5,518.7
COD (mg/l)	188,333.3	169,400	201,200	16,745.6
Total coliforms (cfu/100 mL)	128 × 10^6^	124 × 10^6^	132 × 10^6^	4 × 10^6^
Total helminth (eggs/gTS)	458.00	360.00	556.00	98.00

### 3.2 Effects of chemical additives on physicochemical properties of faecal sludge

#### 3.2.1 Temperature

**Figure 1:**
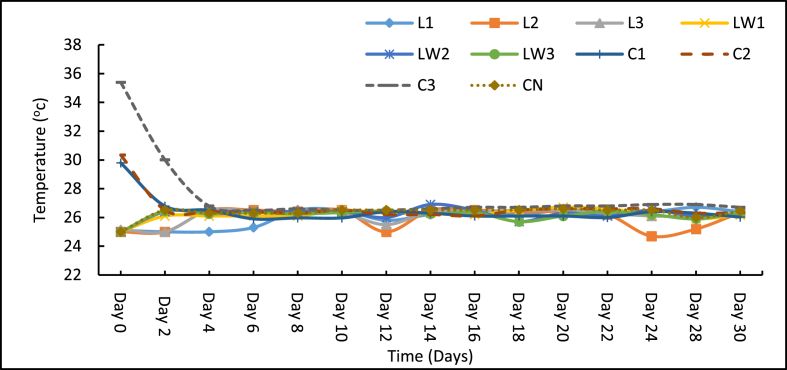
Mean Temperature of samples over 30 days. Note: L1 = 25 mL stock LSEC, L2 = 50 mL stock LSEC, L3 = 75 mL stock LSEC, LW1 = 0.1% DF, LW2 = 0.5% DF, LW3 = 1% DF, C1 = 5 g calcium carbide, C2 = 8 g calcium carbide, C3 = 10 g calcium carbide, CN = control.

The trend of faecal sludge temperature after additive application over the 30 days period is shown in Figure 1. The temperature measured ranged from 24.7 °C to 35.4 °C. The temperature graphs obtained from the study at the initial days of the experiment clearly depicted two different temperature profiles between the calcium carbide treatments (C1–C3) and the rest including the control. There was a noticeable increase in temperature between 29.8 and 35.4 °C on day 0 in the calcium carbide treatments (C1, C2 and C3) and the highest was observed in the treatment with the highest calcium carbide dose (C3). Calcium carbide is noted to be associated with exothermic reactions in water moist environments and this contributed to the rise in temperature [40]. However, the temperature gradually decreased to ambient temperature (25 °C) on day 4 and this remained relatively constant throughout the experiment. The LSEC treatments (L1, L2, L3, LW1, LW2 and LW3) showed a slight increase in temperature (25–26.4 °C) on day 2 after which the temperatures became almost stable at the ambient level for the remaining days of the experiment. The slight rise in temperature upon adding the LSEC additive suggested a probable exothermic reaction at a more lower levels unlike the case of carbide treatments. Meanwhile, a near–constant ambient temperature trend was observed for the control sample, an observation that suggests that no special reactions occurred unlike the additive treatments. In comparison, the calcium carbide treatments generated significantly (p < 0.05) high temperatures than the rest of the treatments on the first day. As already indicated, the relatively high temperature with carbide treatments is concomitant of the heat emission resulting from the calcium carbide and water (moisture content) exothermic reactions as illustrated in Eq. (1) [40, 41].

(1) $${\text{CaC}}_{2\left(\text{s}\right)}+2{\text{H}}_{2}{\text{O}}_{\left(\text{l}\right)}\to {\text{Ca}\left(\text{OH}\right)}_{2\left(\text{s}\right)}+{\text{ C}}_{2}{\text{H}}_{2\left(\text{g}\right)}\text{ T}\;\left[41\right]$$

#### 3.2.2 pH

**Figure 2:**
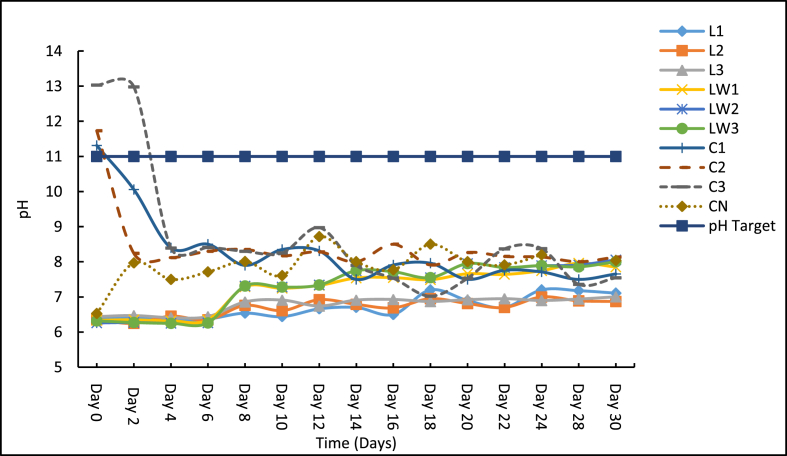
Mean pH of samples over 30 days. Note: L1 = 25 mL stock LSEC, L2 = 50 mL stock LSEC, L3 = 75 mL stock LSEC, LW1 = 0.1% DF, LW2 = 0.5% DF, LW3 = 1% DF, C1 = 5 g calcium carbide, C2 = 8 g calcium carbide, C3 = 10 g calcium carbide, CN = control.

The mean pH values of the treatments ranged from 6.4 to 13. A graph depicting the variation in pH over the experimental period is shown in Figure 2. The results showed changes in the pH levels of the faecal sludge after applying the additives in the treatments (Figure 2). For instance, the pH of the calcium carbide treatments changed immediately from acidic to alkaline and stayed above a pH of 8 from day 0 to day 4. This observation was more pronounced in treatments with high doses of the calcium carbide. Thus, the treatment unit C3 (containing 10 g of calcium carbide) recorded the highest mean pH of 13, followed by C2 (containing 8 g of calcium carbide) with pH of 11.7, and then C1 (containing 5 g of calcium carbide) with mean pH of 11.3. The finding corroborates other studies [6, 42, 43] that positive correlation exist between additive dosage and pH levels depending on the nature of additive and resulting associated reaction products, whether alkaline or acidic. By day 4 into the experiment, the pH of the calcium carbide treatments had declined to 8.1–8.4 and stabilized around that range. The increase in pH values recorded for the carbide treatment during the initial stages of the study could be attributed to the generation of alkaline compounds like hydroxides from calcium carbide reactions with organic substances in the sludge [41] as shown in Eq. (2).

(2) $${\text{C}}_{6}{\text{H}}_{12}{\text{O}}_{6}+7{\text{O}}_{2}+{\text{ CaC}}_{2}\to {\text{Ca}\left(\text{OH}\right)}_{2}+8{\text{CO}}_{2}+2{\text{H}}_{2}\text{O}+3{\text{H}}_{2}\;\left[41\right]$$

Meanwhile, the addition of the LSEC additives to sludge did not cause any obvious change in the pH over the experimental period. But the pH of all the LSEC treated sludge (both stock and diluted treatments) became slightly acidic with the additives due to the acidic nature of the active agent lambda–cyhalothrin according to literature [44, 45].

Generally, the pH pattern after day 4 became almost similar for the treatments and control (Figure 2**)**. Whiles all the LSEC treatments and the control (CN) had some slight increases in pH beyond day 4, all the calcium carbide treatments actually showed stark pH declined (Figure 2). The observation with the calcium carbide treatments could be partly due to volatilization of ammonia at these periods as the glass jars were kept open throughout the experiment [6]. Studies by Gulyas et al., [46] and Patoczka and Wilson [47] have shown that desorption of ammonia occurs when sludge comes into contact with large volumes of air and eventually results in decreased pH. Moreover, respiration of microorganisms present in the sludge, according to Wurst, [48] produces carbon dioxide, which eventually increases carbonic acid concentration thereby contributing to the drop in pH values. Thus, the calcium carbide treatments are associated with the highest increase in pH, followed by the diluted LSEC treatments, and then the stock LSEC treatments.

### 3.3 Key sludge parameters monitored under additive treatment

For the key parameters namely moisture content (MC), BOD_5_, COD, total coliforms (TC) and helminth eggs (HE), the MANOVA tests (see Supplementary Table A1) showed significant influence from the treatment types [Pillai's Trace = 3.310, F (45, 500) = 21.753, p < 0.001], treatment period in weeks [Pillai's Trace = 1.711, F (20, 396) = 14.801, p < 0.001], and the interactions between treatment type and period of treatment in weeks [Pillai's Trace = 2.676, F (180, 500) = 3.198, p < 0.001]. This observation was largely consistent with the Between-Subjects Effects tests (see Supplementary Table A2) except for BOD_5_ and COD which showed no significant effect from the interaction between treatment and the period (p = 0.997 and p = 0.364).

#### 3.3.1 Moisture content

**Figure 3:**
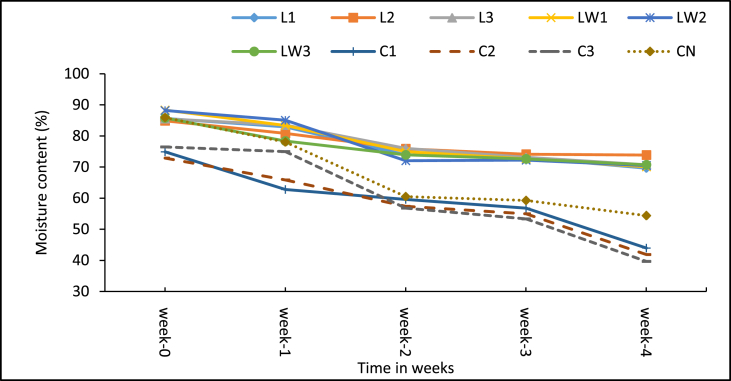
Mean moisture content of samples over a 4–week period. Note: L1 = 25 mL stock LSEC, L2 = 50 mL stock LSEC, L3 = 75 mL stock LSEC, LW1 = 0.1% DF, LW2 = 0.5% DF, LW3 = 1% DF, C1 = 5 g calcium carbide, C2 = 8 g calcium carbide, C3 = 10 g calcium carbide, CN = control.

The average moisture content (MC) of the various treatments at the start of the experiment (week 0) ranged between 72.9% to 88.3% (see Figure 3). Sludge dosed with diluted LSEC had slightly high moisture content – treatment LW1 (0.1% DF) had the highest MC (88.3%), followed by LW2 (0.5% DF) with MC 88.2%, and LW3 (1% DF) with 85.8%. This obviously is because the additive was diluted with water. As expected, the calcium carbide treatments recorded the least MC of 72.9%–76.45%, and these MC values were significantly lower than other treatments including the control (p < 0.001, see Multiple comparison test – Supplementary Table A3). Moreover, a gradual weekly reduction in moisture content was observed for all the treatments over the experimental period, and the calcium carbide treatments remained the species with the lowest MC of all treatments (see Figure 3). The low moisture content measured for the calcium carbide treatments could be attributed partly to the hydrolytic chemical reaction which generates acetylene (C_2_H_2_) gas and heat to aid evaporation and dehydration [40] (see Eq. (1)). The LSEC treatments consistently recorded high levels of moisture content throughout the experiment because it was in solution form and undeniably contributed to the high moisture content. It was also observed that the LSEC additive caused some form of sludge dissolution in the respective treatment units. The more dissolved sludge from LSEC treatments remained in the setups because of the impermeable glass jars, which otherwise in real toilet pits the apparent “liquid” sludge would have its water content infiltrated into the surrounding soil [3]. This phenomenon could be the probable reason for the claim and promotion among some artisans and residents that LSEC works as toilet additive. A field trial with toilet pits will be required to test such an assumption in subsequent studies as the current study could not consider that aspect. Meanwhile, the average moisture content for the control was reduced by about 32%, which was lower and outside the range 50–90% MC reduction quoted in literature for sludge under natural decomposition [3, 5, 18]. The lower MC reduction in the current study could be due to the short decomposition period of 30 days.

#### 3.3.2 Mass reduction

**Figure 4:**
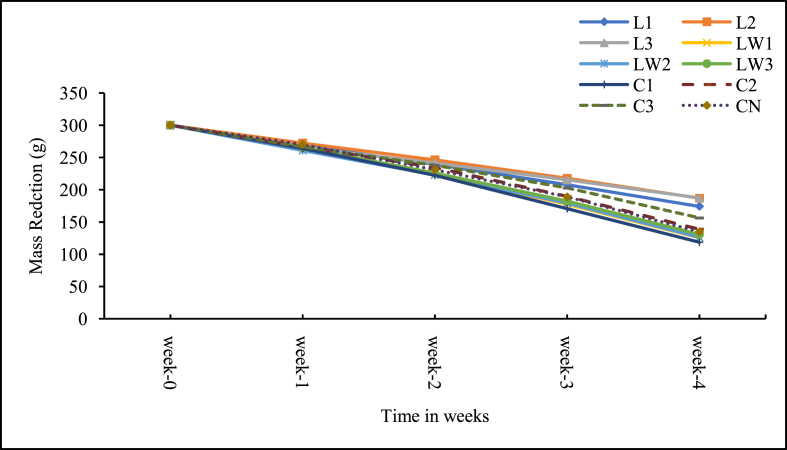
Mass loss in sludge samples. Note: L1 = 25 mL stock LSEC, L2 = 50 mL stock LSEC, L3 = 75 mL stock LSEC, LW1 = 0.1% DF, LW2 = 0.5% DF, LW3 = 1% DF, C1 = 5 g calcium carbide, C2 = 8 g calcium carbide, C3 = 10 g calcium carbide, CN = control.

**Figure 5:**
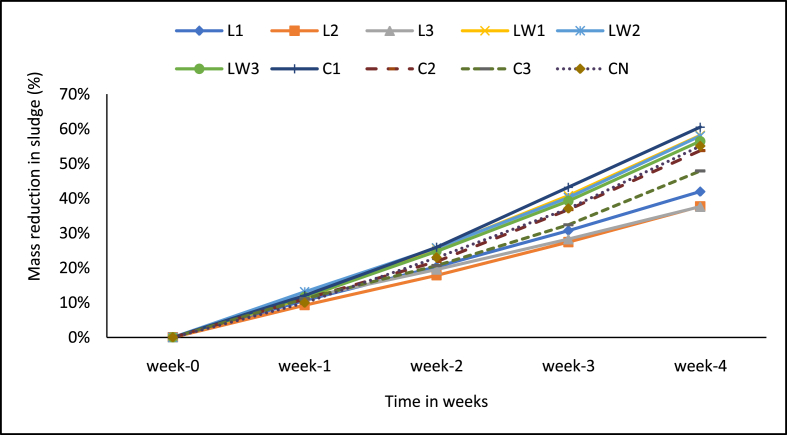
Percentage mass loss in sludge samples. Note: L1 = 25 mL stock LSEC, L2 = 50 mL stock LSEC, L3 = 75 mL stock LSEC, LW1 = 0.1% DF, LW2 = 0.5% DF, LW3 = 1% DF, C1 = 5 g calcium carbide, C2 = 8 g calcium carbide, C3 = 10 g calcium carbide, CN = control.

The mass loss and percentage reduction are presented in Figures 4 and 5 respectively. In all the treatments including the control, the mass of faecal sludge in each experimental unit decreased with time (Figure 5). The mass of the faecal sludge on the average dropped from 300 g to 125 g. The mass loss at the end of the experiment was greatest (61%) in the calcium carbide treatment dosed with 5 g of the additive (C1), followed by two of the diluted LSEC treatments (0.1% DF, LW1; and 0.5% DF, LW2) with 58% each, then 1%DF (LW3) with 56% and the control (CN) with 55%. Those with low mass reductions below 50% were the stock LSEC treatments (38%–42%) and the 10 g calcium carbide treatment (C3) with 48%. The cause and dynamics of the mass loss due to the additives are not clearly understood here and will need separate further studies. However, the low mass loss for the stock LSEC treatments could partly be ascribed to increasing less biodegradable organics from the additive (as probably seen in the high COD), and liquid additive less evaporative water loss. On the other hand, mass loss achieved by the 5 g calcium carbide treatment (C1) could be due to a probable optimum biochemical degradation that was aided by a comparatively moderate quantity of the carbide additive used. In this case, the complex relationship among temperature, moisture content, pH and biodegradation processes could have partly enhanced microbial decompositions of faecal sludge as well [1]. The mass loss in the control may also be largely due to dehydration (loss in moisture content), and also biochemical degradation of faecal sludge content [17]. Buckley et al., [18] assert that faecal sludge mass losses could be linked to dehydration and biological activities especially under favourable conditions. Also, alkaline hydrolyses under high pH conditions contribute to mass reduction under both aerobic and anaerobic conditions [8, 10, 12]. Largely, the mass losses achieved in this study were higher than those recorded by Awere and Edu–Buandoh [3] (6.6–7.3%). The rates of mass reduction in our current study are higher than the 30% reported for aerobic digestion and somehow not too far from the 70% reported for anaerobic digestion [49]. Comparatively, this is obviously a promising result from a limited study period which is also close to frequent period of desludging the sludge sources, the public toilets.

#### 3.3.3 Biochemical and chemical oxygen demand

**Figure 6:**
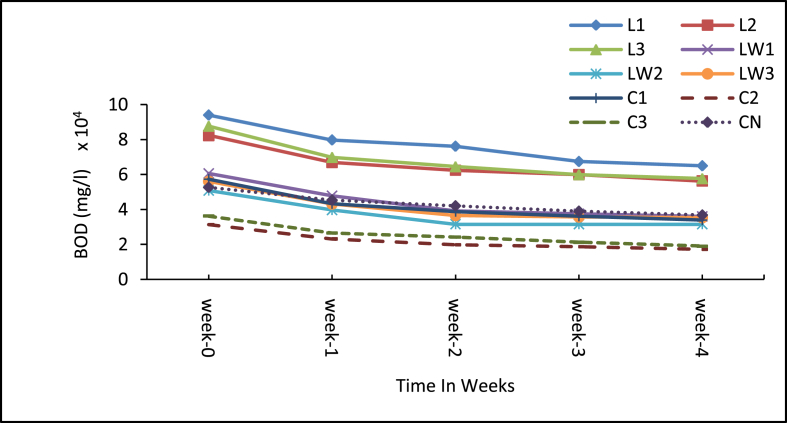
Mean BOD_5_ levels of treatment samples. Note: L1 = 25 mL stock LSEC, L2 = 50 mL stock LSEC, L3 = 75 mL stock LSEC, LW1 = 0.1% DF, LW2 = 0.5% DF, LW3 = 1% DF, C1 = 5 g calcium carbide, C2 = 8 g calcium carbide, C3 = 10 g calcium carbide, CN = control.

**Figure 7:**
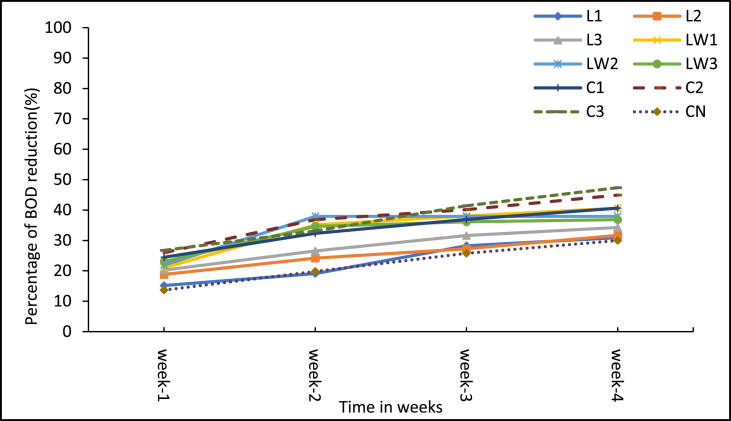
Cumulative mean reduction in BOD_5_ levels. Note: L1 = 25 mL stock LSEC, L2 = 50 mL stock LSEC, L3 = 75 mL stock LSEC, LW1 = 0.1% DF, LW2 = 0.5% DF, LW3 = 1% DF, C1 = 5 g calcium carbide, C2 = 8 g calcium carbide, C3 = 10 g calcium carbide, CN = control.

Figure 6 shows the weekly changes in BOD_5_ levels in the sludge over the treatment period. The calcium carbide treatments declined in BOD_5_ levels throughout the experimental period and the lowest BOD_5_ of 17,252 mg/L came from the medium dose of 8g (C2). This in part corroborates the findings by Chukwu [41] that calcium carbide reduces the BOD of faecal sludge and the reduction increases with increased carbide dosage to a certain level. The BOD_5_ reduction could be due to consumptive reaction with organic matter in the sludge. In terms of percentage reduction, Figure 7 shows the cumulative weekly reduction in BOD_5_ for all the treatments and the control. Overall, the calcium carbide treatments had the highest reduction rates especially with the highest dose of 10 g (C3) recording 47.4%, followed by medium dose of 8 g (C2) with 44.9%. The control (CN) had the lowest (30%) reduction rate and this was expected because no agent was added to boost any biochemical treatment processes. The results showed an upsurge in BOD_5_ with the stock LSEC additive treatments. These high BOD_5_ levels were observed throughout the experiment. All these observations suggest that the additives contributed some influence on the BOD_5_ reduction in the treatments at some point, and the most conspicuous influence is seen from the calcium carbide. Among the LSEC treatments, LW1 (0.1% DF) had the highest BOD_5_ reduction of 40.6%, probably because the additive was more effective at such low dilution level. Generally, significant differences exist between the initial and final mean BOD_5_ values of all the treatments including the control (p < 0.05). In comparison, the 8 g and 10 g calcium carbide treatments and the stock LSEC treatments had higher BOD_5_ levels than the control and these differences were significant (p < 0.001, see Supplementary Table A3). Meanwhile, difference between the control and the diluted LSEC treatment LW2 (0.5% DF) was significant (p = 0.010) in favour of the diluted LSEC with low BOD_5_ loads. This notwithstanding, the final BOD_5_ concentrations of all the treatments including the control were about 1000 times far above the recommended Ghana EPA value (50 mg/L) for discharge into the environment.

**Figure 8:**
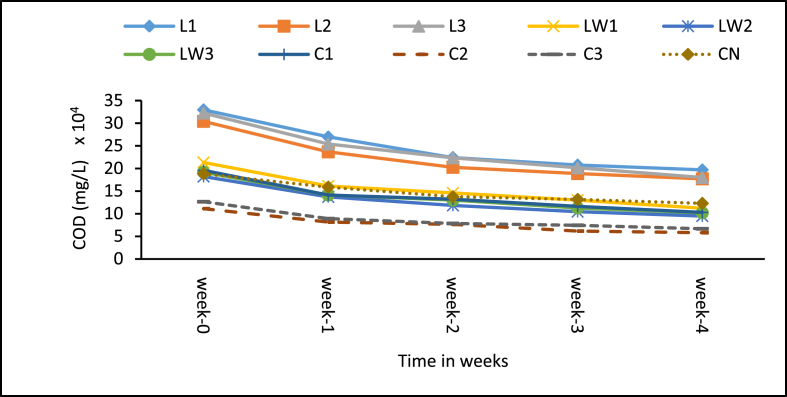
Mean COD levels of treatment samples. Note: L1 = 25 mL stock LSEC, L2 = 50 mL stock LSEC, L3 = 75 mL stock LSEC, LW1 = 0.1% DF, LW2 = 0.5% DF, LW3 = 1% DF, C1 = 5 g calcium carbide, C2 = 8 g calcium carbide, C3 = 10 g calcium carbide, CN = control.

**Figure 9:**
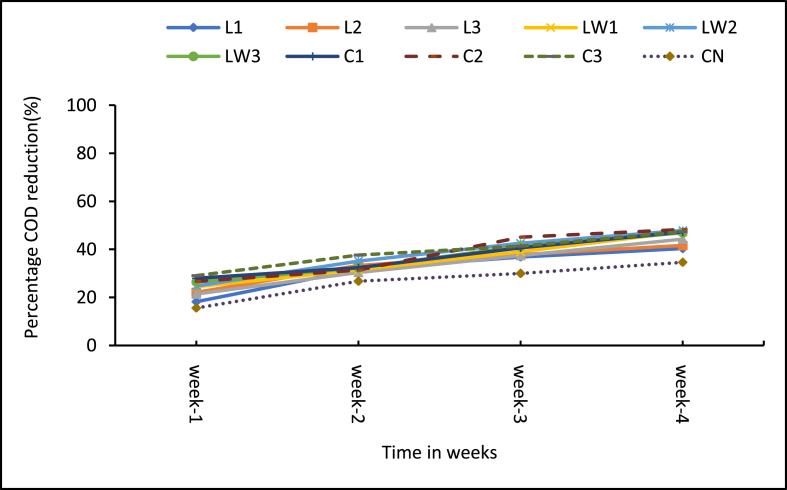
Cumulative mean reduction in COD levels. Note: L1 = 25 mL stock LSEC, L2 = 50 mL stock LSEC, L3 = 75 mL stock LSEC, LW1 = 0.1% DF, LW2 = 0.5% DF, LW3 = 1% DF, C1 = 5 g calcium carbide, C2 = 8 g calcium carbide, C3 = 10 g calcium carbide, CN = control.

The COD values for the treatments including the control are presented in Figure 8. Higher initial COD levels were associated with stock LSEC treatment, an indication that the additive contributed some form of COD. The COD patterns of treatments as depicted in Figure 8 is similar to the BOD_5_ trend discussed earlier. Generally, all the treatments including the control experienced gradual weekly decline in COD levels over the period of the experiment (Figure 9). Cumulatively, COD reduction rate was high for the calcium carbide treatments with the medium dose of 8 g (C2) having 48.3%. The control (CN) had the lowest COD reduction of 34.7%. As clearly shown among the LSEC treatments, the diluted ones consistently recorded higher weekly COD reduction rates with LW2 (0.5% DF) recording the highest reduction of 47.9% (see Figure 8). The Least significant difference (LCD) multiple comparison tests showed significant differences between the control and most treatments (6 out of 9) (p < 0.001, see Supplementary Table A3). Generally, low reduction performance came from LSEC stock, and by this the additive most likely introduced additional COD from the onset. Comparatively better COD reduction is associated with carbide additive. However, none of the treatments were efficient to reduce COD levels anywhere near the regulatory discharge limit (250 mg/L set by Ghana EPA) for wastewater.

#### 3.3.4 Microbial loads

**Figure 10:**
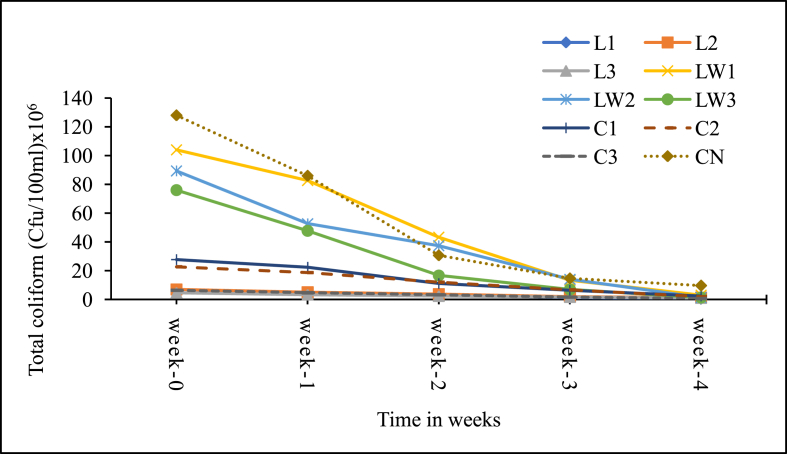
Mean Total coliform levels in samples. Note: L1 = 25 mL stock LSEC, L2 = 50 mL stock LSEC, L3 = 75 mL stock LSEC, LW1 = 0.1% DF, LW2 = 0.5% DF, LW3 = 1% DF, C1 = 5 g calcium carbide, C2 = 8 g calcium carbide, C3 = 10 g calcium carbide, CN = control.

**Figure 11:**
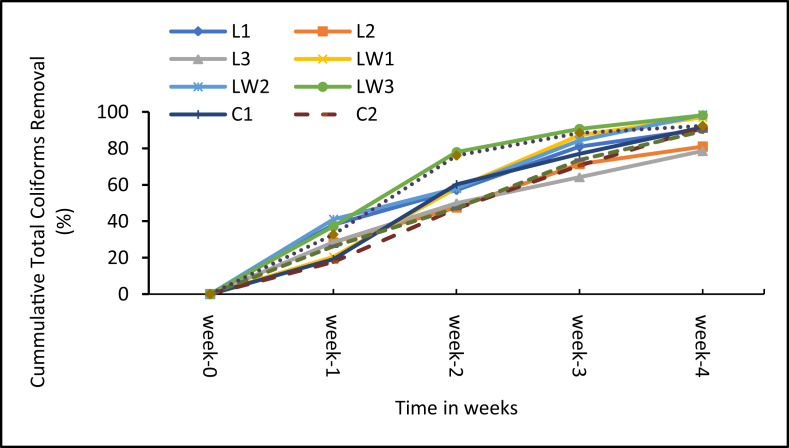
Mean Total Coliform percentage removal. Note: L1 = 25 mL stock LSEC, L2 = 50 mL stock LSEC, L3 = 75 mL stock LSEC, LW1 = 0.1% DF, LW2 = 0.5% DF, LW3 = 1% DF, C1 = 5 g calcium carbide, C2 = 8 g calcium carbide, C3 = 10 g calcium carbide, CN = control.

**Figure 12:**
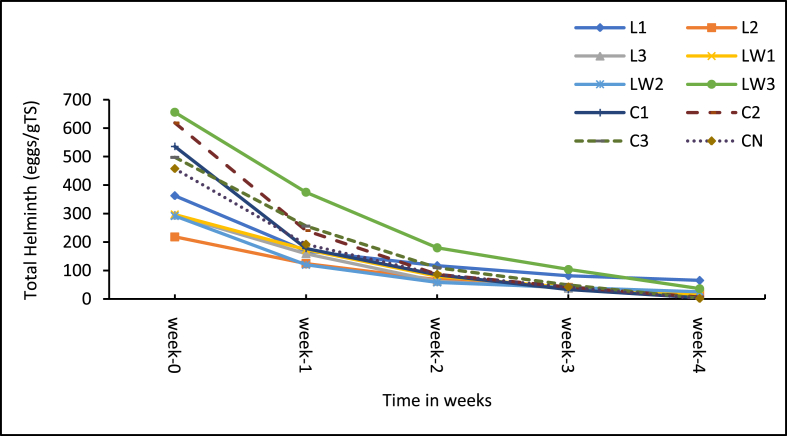
Total helminth egg levels in treatments. Note: L1 = 25 mL stock LSEC, L2 = 50 mL stock LSEC, L3 = 75 mL stock LSEC, LW1 = 0.1% DF, LW2 = 0.5% DF, LW3 = 1% DF, C1 = 5 g calcium carbide, C2 = 8g calcium carbide, C3 = 10 g calcium carbide, CN = control.

**Figure 13:**
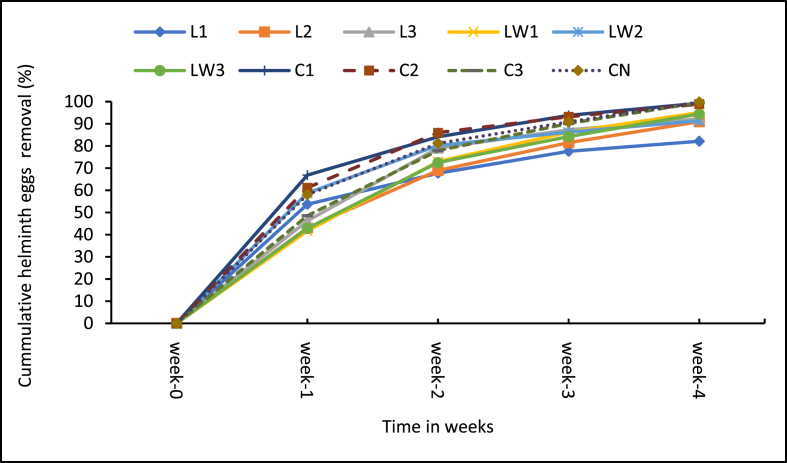
Total helminth egg removal percentage. Note: L1 = 25 mL stock LSEC, L2 = 50 mL stock LSEC, L3 = 75 mL stock LSEC, LW1 = 0.1% DF, LW2 = 0.5% DF, LW3 = 1% DF, C1 = 5 g Calcium carbide, C2 = 8 g calcium carbide, C3 = 10 g calcium carbide, CN = control.

The microbial content is important for public health and environmental acceptability of the additive treated sludge. Figure 10 shows the weekly change in total coliform (TC) content for the treatments and control whiles Figure 11 presents the corresponding percentage reduction rates. Likewise, Figure 12 shows the weekly change in helminth eggs content in the treatments and control, and the respective reductions rates are presented in Figure 13.

From Figure 10, slight variation in TC load was observed among treatments and the control immediately after additives application (Figure 10). For instance, the difference between the control and LSEC treatments (both stock and diluted) was no less than 24 × 10^6^ cfu/100 mL TC load at the initial stage of the experiment, whiles that between the control and carbide treatments was no less than 100 × 10^6^ cfu/100 mL TC. Such differences in TC loads in the case of the control and the LSEC treatments could be influenced by the bactericidal effect of LSEC on microorganisms. The observed differences in TC loads between the control and calcium carbide during the early stages could be linked to the rise in pH of the carbide treated sludge above 11 which is detrimental to microbial survival. Among the treatments, TC concentration at the end of the experiment was lowest with the stock LSEC treatments, followed by the calcium carbide treatments and the diluted LSEC treatments. The control had the highest TC load and the differences between the treatments and control were significant (p < 0.001). But in terms of TC die–off rates, the highest was recorded with the diluted LSEC treatments (97%–98.5%), followed by the calcium carbide treatments (89%–92%) and the control (92%). The stock LSEC treatments had the lowest die-off rates (79%–90%). This observation suggests that the diluted LSEC is somehow effective than the stock likely due to ease of active agent release and high microbial vulnerability in aqueous environment [50]. The general weekly decline in TC concentrations across treatments (Figure 10) is consistent with the findings by Kemboi et al., [1] who largely attributed high die-off rates to high pH levels, favouring sludge sanitization. Our results further confirm the findings by Pecson et al., [51] and Ouali et al., [52] who observed that high pH levels (pH > 8.5) among other factors lead to decline in coliform loads in sludge. This probably explains why the stock LSEC treatments (with the least pH values < 7.5 throughout the study) gave high total coliform counts.

The other environmental conditions favouring sludge sanitization with the diluted LSEC treatments could include availability of active disinfectant or bactericidal agents like the aromatic, alcohol and sulphonate constituents [28, 53]. Nevertheless, none of the treatments including the control could sanitise the faecal sludge to the microbial levels recommended by Ghana EPA (400 cfu/100 mL). The treated sludge under such conditions need serious treatment attention before any means of disposal and/or potential use for agricultural purposes.

With regards to helminth eggs (HE), the total concentration in the beginning of the study ranged from 218 to 656 eggs/gTS. The helminth egg counts and the trends observed are similar to those reported by Appiah–Effah et al., [54, 55]. A sharp decline in helminth egg loads was observed in all treatments by week 1 (Figure 12) and subsequently declined slowly as observed by Appiah–Effah et al. [54], in their sludge composting study. In this study, the reduction in helminth eggs could be attributed to the combined effects of temperature, pH and moisture content levels which influence die–off rates especially through desiccation [9, 51, 56]. At the end of the experiment, the control (CN) had the lowest helminth egg count of 1 egg/g/TS, followed by the calcium carbide treatments (C1, C2 and C3) with loads of 2–7 eggs/gTS. All the LSEC treatments had high helminth eggs counts ranging from 15–36 eggs/gTS for the diluted LSEC treatment and 20–65 eggs/gTS for the stock LSEC treatments. While the helminth egg reduction in treatments was expected, it is unclear why the control had the highest die–off (see Figure 13). Helminth egg die–off under natural decomposition processes among other factors is influenced by temperature, pH and moisture content [56, 57]. But the die–off due to the control could probably be due to uneven distribution of the eggs than expected during sampling. In the case of the carbide treatments (C1, C2 and C3), the egg counts reduction could be attributed to the synergistic performance of high pH [1, 11] and lower moisture content that was exhibited by the powdery additive [54].

From the MANOVA multiple comparison test, there is significant differences in the helminth eggs (HE) concentration between the control and 5 out of 9 (55%) of the treatments (see Supplementary Table A3). Only three of LSEC treatments performed significantly better than the control, namely L2 and L3 (stock) and LW2 (dilute). In general, the two additives did not achieve the expected level of sanitised sludge (low TC and helminth egg loads) at the end of the 30 days period contrary to the assertion by Mamani et al., [11] that chemical additives are capable of rapid sanitisation (within 2 weeks). Moreover, the additives comparatively did influence the rate of HE reduction in a significantly positive direction for the three LSEC treatments already stated while all the carbide treatments performed poorly with dilute LSEC LW3 (Lw 1%).

## 4 Conclusion

The study showed that faecal sludge from the public toilets was slightly acidic with high contents of moisture and microbial loads as expected, and also high levels slowly degradable organic matter, thus more COD than BOD_5_ giving it a lower biodegradability property. The two additives tested especially calcium carbide showed some influence on the faecal sludge physico–chemical properties like pH and temperature increase or decrease immediately at the early stages of treatment but stabilized shortly afterwards to ambient conditions. Statistically, the additives could significantly treat the faecal sludge to appreciable levels by moisture content reduction between 39 – 74%, BOD_5_ (30–47%), total coliform (79–98.5%) and helminth eggs (82–99.6%). The carbide additive could be applied best for the achievement of lowering moisture content and organic matter (BOD_5_ & COD) in faecal sludge treatment. On the other hand, stock LSEC could be applied for lowering microbial loads (both total coliforms and helminth eggs, and dilute LSEC (0.5% DF) could give some BOD_5_ & COD reductions. However, the two additives would not treat faecal sludge to the levels that meet Ghana's Environmental Protection Agency discharge limits. Given that this topic is still a grey research area, more studies especially on the mechanisms by which the additives treat (by mass loss, moisture content loss, decomposition and sanitization) is highly recommended. In addition, this study should be piloted in a real–world toilet pits to allow for the testing of the current assumptions and conclusions.

## Declarations

### Author contribution statement

Eugene Appiah-Effah: Conceived and designed the experiments; Performed the experiments; Analyzed and interpreted the data; Wrote the paper.

Godwin Armstrong Duku: Conceived and designed the experiments; Analyzed and interpreted the data; Contributed reagents, materials, analysis tools or data; Wrote the paper.

Bismark Dwumfour-Asare: Conceived and designed the experiments; Analyzed and interpreted the data; Wrote the paper.

Isaac Manu: Performed the experiments; Contributed reagents, materials, analysis tools or data.

Kwabena Biritwum Nyarko: Conceived and designed the experiments; Analyzed and interpreted the data; Wrote the paper.

### Funding statement

This work was supported by the Regional Water and Environmental Sanitation Centre, Kumasi (RWESCK) at the Kwame Nkrumah University of Science and Technology, Kumasi with funding from Ghana Government and the World Bank under the Africa Centre's of Excellence project.

### Competing interest statement

The authors declare no conflict of interest.

### Additional information

No additional information is available for this paper.

## Appendix A Supplementary data

Supplementary Table A1.docx Supplementary Table A1.docx

Supplementary Table A2.docx Supplementary Table A2.docx

Supplementary Table A3.docx Supplementary Table A3.docx

Supplementary Table A4.docx Supplementary Table A4.docx
