# Supplementary material for: Toilet chemical additives and their effect on faecal sludge characteristics
Source: Heliyon. 2020 Sep 23;6(9):e04998. doi: 10.1016/j.heliyon.2020.e04998 (PMC7511817; doi:10.1016/j.heliyon.2020.e04998)
Supplement: Supplementary Table A1.docx [file mmc1.docx]

| Table A1. Multivariate Tests^a^ | | | | | | |
| --- | --- | --- | --- | --- | --- | --- |
| Effect | | Value | F | Hypothesis df | Error df | Sig. |
| Intercept | Pillai's Trace | 1.000 | 40326.890^b^ | 5.000 | 96.000 | .000 |
|  | Wilks' Lambda | .000 | 40326.890^b^ | 5.000 | 96.000 | .000 |
|  | Hotelling's Trace | 2100.359 | 40326.890^b^ | 5.000 | 96.000 | .000 |
|  | Roy's Largest Root | 2100.359 | 40326.890^b^ | 5.000 | 96.000 | .000 |
| Treatment Types | Pillai's Trace | 3.310 | 21.753 | 45.000 | 500.000 | .000 |
|  | Wilks' Lambda | .000 | 64.611 | 45.000 | 432.534 | .000 |
|  | Hotelling's Trace | 67.487 | 141.572 | 45.000 | 472.000 | .000 |
|  | Roy's Largest Root | 36.085 | 400.948^c^ | 9.000 | 100.000 | .000 |
| Week | Pillai's Trace | 1.711 | 14.801 | 20.000 | 396.000 | .000 |
|  | Wilks' Lambda | .006 | 57.075 | 20.000 | 319.346 | .000 |
|  | Hotelling's Trace | 59.345 | 280.406 | 20.000 | 378.000 | .000 |
|  | Roy's Largest Root | 57.999 | 1148.388^c^ | 5.000 | 99.000 | .000 |
| Treatment Type * Week | Pillai's Trace | 2.676 | 3.198 | 180.000 | 500.000 | .000 |
|  | Wilks' Lambda | .002 | 6.461 | 180.000 | 481.579 | .000 |
|  | Hotelling's Trace | 28.102 | 14.738 | 180.000 | 472.000 | .000 |
|  | Roy's Largest Root | 21.487 | 59.687^c^ | 36.000 | 100.000 | .000 |
| a. Design: Intercept + Treatment Type + Week + Treatment Type * Week | | | | | | |
| b. Exact statistic | | | | | | |
| c. The statistic is an upper bound on F that yields a lower bound on the significance level. | | | | | | |
