# Supplementary material for: Toilet chemical additives and their effect on faecal sludge characteristics
Source: Heliyon. 2020 Sep 23;6(9):e04998. doi: 10.1016/j.heliyon.2020.e04998 (PMC7511817; doi:10.1016/j.heliyon.2020.e04998)
Supplement: Supplementary Table A2.docx [file mmc2.docx]

| **Table A2 Tests of Between-Subjects Effects** | | | | | | |
| --- | --- | --- | --- | --- | --- | --- |
| a. R Squared = .928 (Adjusted R Squared = .893) | | Type III Sum of Squares | df | Mean Square | F | Sig. |
| Corrected Model | BOD5 | 53776281884.167^a^ | 49 | 1097475140.493 | 26.290 | .000 |
|  | COD | 657238027884.000^b^ | 49 | 13413020977.225 | 28.599 | .000 |
|  | moisture content | 21491.383^c^ | 49 | 438.600 | 108.234 | .000 |
|  | total coliforms | 137884.167^d^ | 49 | 2813.963 | 143.228 | .000 |
|  | helminth eggs | 4037489.093^e^ | 49 | 82397.737 | 30.377 | .000 |
| Intercept | BOD5 | 325024668588.167 | 1 | 325024668588.167 | 7786.085 | .000 |
|  | COD | 3696976349440.670 | 1 | 3696976349440.67 | 7882.589 | .000 |
|  | moisture content | 757293.532 | 1 | 757293.532 | 186879.344 | .000 |
|  | total coliforms | 67204.167 | 1 | 67204.167 | 3420.640 | .000 |
|  | helminth eggs | 3699234.240 | 1 | 3699234.240 | 1363.780 | .000 |
| Treatment Type | BOD5 | 43760369559.633 | 9 | 4862263284.404 | 116.477 | .000 |
|  | COD | 470731205231.600 | 9 | 52303467247.956 | 111.520 | .000 |
|  | moisture content | 9908.703 | 9 | 1100.967 | 271.689 | .000 |
|  | total coliforms | 54710.433 | 9 | 6078.937 | 309.413 | .000 |
|  | helminth eggs | 372868.027 | 9 | 41429.781 | 15.274 | .000 |
| week | BOD5 | 9353476554.333 | 4 | 2338369138.583 | 56.016 | .000 |
|  | COD | 168146981383.667 | 4 | 42036745345.917 | 89.630 | .000 |
|  | moisture content | 9741.453 | 4 | 2435.363 | 600.981 | .000 |
|  | total coliforms | 42137.200 | 4 | 10534.300 | 536.188 | .000 |
|  | helminth eggs | 3201143.693 | 4 | 800285.923 | 295.038 | .000 |
| Treatment Type * Week | BOD5 | 662435770.200 | 36 | 18400993.617 | .441 | .997 |
|  | COD | 18359841268.733 | 36 | 509995590.798 | 1.087 | .364 |
|  | moisture content | 1841.226 | 36 | 51.145 | 12.621 | .000 |
|  | total coliforms | 41036.533 | 36 | 1139.904 | 58.020 | .000 |
|  | helminth eggs | 463477.373 | 36 | 12874.371 | 4.746 | .000 |
| Error | BOD5 | 4174430122.667 | 100 | 41744301.227 |  |  |
|  | COD | 46900531639.333 | 100 | 469005316.393 |  |  |
|  | moisture content | 405.231 | 100 | 4.052 |  |  |
|  | total coliforms | 1964.667 | 100 | 19.647 |  |  |
|  | helminth eggs | 271248.667 | 100 | 2712.487 |  |  |
| Total | BOD5 | 382975380595.000 | 150 |  |  |  |
|  | COD | 4401114908964.000 | 150 |  |  |  |
|  | moisture content | 779190.146 | 150 |  |  |  |
|  | total coliforms | 207053.000 | 150 |  |  |  |
|  | helminth eggs | 8007972.000 | 150 |  |  |  |
| Corrected Total | BOD5 | 57950712006.833 | 149 |  |  |  |
|  | COD | 704138559523.333 | 149 |  |  |  |
|  | moisture content | 21896.614 | 149 |  |  |  |
|  | total coliforms | 139848.833 | 149 |  |  |  |
|  | helminth eggs | 4308737.760 | 149 |  |  |  |
| a. R Squared = .928 (Adjusted R Squared = .893) | | | | | | |
| b. R Squared = .933 (Adjusted R Squared = .901) | | | | | | |
| c. R Squared = .981 (Adjusted R Squared = .972) | | | | | | |
| d. R Squared = .986 (Adjusted R Squared = .979) | | | | | | |
| e. R Squared = .937 (Adjusted R Squared = .906) | | | | | | |
|  |  |  |  |  |  |  |
|  |  |  |  |  |  |  |
| Post Hoc Tests |  |  |  |  |  |  |
|  |  |  |  |  |  |  |
|  |  |  |  |  |  |  |
| treatment types |  |  |  |  |  |  |
