# Supplementary material for: Toilet chemical additives and their effect on faecal sludge characteristics
Source: Heliyon. 2020 Sep 23;6(9):e04998. doi: 10.1016/j.heliyon.2020.e04998 (PMC7511817; doi:10.1016/j.heliyon.2020.e04998)
Supplement: Supplementary Table A3.docx [file mmc3.docx]

**Table A3. Multiple Comparisons**

| Dependent Variable | | | Mean Difference (I-J) | Std. Error | Sig. | 95% Confidence Interval | |
| --- | --- | --- | --- | --- | --- | --- | --- |
|  |  |  |  |  |  | Lower Bound | Upper Bound |
| BOD5 | control | C5g | 1308.467 | 2359.2174 | .580 | -3372.153 | 5989.087 |
|  |  | C8g | 21130.400^*^ | 2359.2174 | .000 | 16449.780 | 25811.020 |
|  |  | C10g | 17738.933^*^ | 2359.2174 | .000 | 13058.313 | 22419.553 |
|  |  | Lw0.1% | -1052.667 | 2359.2174 | .656 | -5733.287 | 3627.953 |
|  |  | Lw0.5% | 6165.000^*^ | 2359.2174 | .010 | 1484.380 | 10845.620 |
|  |  | Lw1% | 1827.000 | 2359.2174 | .441 | -2853.620 | 6507.620 |
|  |  | L25ml | -33289.467^*^ | 2359.2174 | .000 | -37970.087 | -28608.847 |
|  |  | L50ml | -22379.267^*^ | 2359.2174 | .000 | -27059.887 | -17698.647 |
|  |  | L75ml | -24781.400^*^ | 2359.2174 | .000 | -29462.020 | -20100.780 |
|  | C5g | control | -1308.467 | 2359.2174 | .580 | -5989.087 | 3372.153 |
|  |  | C8g | 19821.933^*^ | 2359.2174 | .000 | 15141.313 | 24502.553 |
|  |  | C10g | 16430.467^*^ | 2359.2174 | .000 | 11749.847 | 21111.087 |
|  |  | Lw0.1% | -2361.133 | 2359.2174 | .319 | -7041.753 | 2319.487 |
|  |  | Lw0.5% | 4856.533^*^ | 2359.2174 | .042 | 175.913 | 9537.153 |
|  |  | Lw1% | 518.533 | 2359.2174 | .826 | -4162.087 | 5199.153 |
|  |  | L25ml | -34597.933^*^ | 2359.2174 | .000 | -39278.553 | -29917.313 |
|  |  | L50ml | -23687.733^*^ | 2359.2174 | .000 | -28368.353 | -19007.113 |
|  |  | L75ml | -26089.867^*^ | 2359.2174 | .000 | -30770.487 | -21409.247 |
|  | C8g | control | -21130.400^*^ | 2359.2174 | .000 | -25811.020 | -16449.780 |
|  |  | C5g | -19821.933^*^ | 2359.2174 | .000 | -24502.553 | -15141.313 |
|  |  | C10g | -3391.467 | 2359.2174 | .154 | -8072.087 | 1289.153 |
|  |  | Lw0.1% | -22183.067^*^ | 2359.2174 | .000 | -26863.687 | -17502.447 |
|  |  | Lw0.5% | -14965.400^*^ | 2359.2174 | .000 | -19646.020 | -10284.780 |
|  |  | Lw1% | -19303.400^*^ | 2359.2174 | .000 | -23984.020 | -14622.780 |
|  |  | L25ml | -54419.867^*^ | 2359.2174 | .000 | -59100.487 | -49739.247 |
|  |  | L50ml | -43509.667^*^ | 2359.2174 | .000 | -48190.287 | -38829.047 |
|  |  | L75ml | -45911.800^*^ | 2359.2174 | .000 | -50592.420 | -41231.180 |
|  | C10g | control | -17738.933^*^ | 2359.2174 | .000 | -22419.553 | -13058.313 |
|  |  | C5g | -16430.467^*^ | 2359.2174 | .000 | -21111.087 | -11749.847 |
|  |  | C8g | 3391.467 | 2359.2174 | .154 | -1289.153 | 8072.087 |
|  |  | Lw0.1% | -18791.600^*^ | 2359.2174 | .000 | -23472.220 | -14110.980 |
|  |  | Lw0.5% | -11573.933^*^ | 2359.2174 | .000 | -16254.553 | -6893.313 |
|  |  | Lw1% | -15911.933^*^ | 2359.2174 | .000 | -20592.553 | -11231.313 |
|  |  | L25ml | -51028.400^*^ | 2359.2174 | .000 | -55709.020 | -46347.780 |
|  |  | L50ml | -40118.200^*^ | 2359.2174 | .000 | -44798.820 | -35437.580 |
|  |  | L75ml | -42520.333^*^ | 2359.2174 | .000 | -47200.953 | -37839.713 |
|  | Lw0.1% | control | 1052.667 | 2359.2174 | .656 | -3627.953 | 5733.287 |
|  |  | C5g | 2361.133 | 2359.2174 | .319 | -2319.487 | 7041.753 |
|  |  | C8g | 22183.067^*^ | 2359.2174 | .000 | 17502.447 | 26863.687 |
|  |  | C10g | 18791.600^*^ | 2359.2174 | .000 | 14110.980 | 23472.220 |
|  |  | Lw0.5% | 7217.667^*^ | 2359.2174 | .003 | 2537.047 | 11898.287 |
|  |  | Lw1% | 2879.667 | 2359.2174 | .225 | -1800.953 | 7560.287 |
|  |  | L25ml | -32236.800^*^ | 2359.2174 | .000 | -36917.420 | -27556.180 |
|  |  | L50ml | -21326.600^*^ | 2359.2174 | .000 | -26007.220 | -16645.980 |
|  |  | L75ml | -23728.733^*^ | 2359.2174 | .000 | -28409.353 | -19048.113 |
|  | Lw0.5% | control | -6165.000^*^ | 2359.2174 | .010 | -10845.620 | -1484.380 |
|  |  | C5g | -4856.533^*^ | 2359.2174 | .042 | -9537.153 | -175.913 |
|  |  | C8g | 14965.400^*^ | 2359.2174 | .000 | 10284.780 | 19646.020 |
|  |  | C10g | 11573.933^*^ | 2359.2174 | .000 | 6893.313 | 16254.553 |
|  |  | Lw0.1% | -7217.667^*^ | 2359.2174 | .003 | -11898.287 | -2537.047 |
|  |  | Lw1% | -4338.000 | 2359.2174 | .069 | -9018.620 | 342.620 |
|  |  | L25ml | -39454.467^*^ | 2359.2174 | .000 | -44135.087 | -34773.847 |
|  |  | L50ml | -28544.267^*^ | 2359.2174 | .000 | -33224.887 | -23863.647 |
|  |  | L75ml | -30946.400^*^ | 2359.2174 | .000 | -35627.020 | -26265.780 |
|  | Lw1% | control | -1827.000 | 2359.2174 | .441 | -6507.620 | 2853.620 |
|  |  | C5g | -518.533 | 2359.2174 | .826 | -5199.153 | 4162.087 |
|  |  | C8g | 19303.400^*^ | 2359.2174 | .000 | 14622.780 | 23984.020 |
|  |  | C10g | 15911.933^*^ | 2359.2174 | .000 | 11231.313 | 20592.553 |
|  |  | Lw0.1% | -2879.667 | 2359.2174 | .225 | -7560.287 | 1800.953 |
|  |  | Lw0.5% | 4338.000 | 2359.2174 | .069 | -342.620 | 9018.620 |
|  |  | L25ml | -35116.467^*^ | 2359.2174 | .000 | -39797.087 | -30435.847 |
|  |  | L50ml | -24206.267^*^ | 2359.2174 | .000 | -28886.887 | -19525.647 |
|  |  | L75ml | -26608.400^*^ | 2359.2174 | .000 | -31289.020 | -21927.780 |
|  | L25ml | control | 33289.467^*^ | 2359.2174 | .000 | 28608.847 | 37970.087 |
|  |  | C5g | 34597.933^*^ | 2359.2174 | .000 | 29917.313 | 39278.553 |
|  |  | C8g | 54419.867^*^ | 2359.2174 | .000 | 49739.247 | 59100.487 |
|  |  | C10g | 51028.400^*^ | 2359.2174 | .000 | 46347.780 | 55709.020 |
|  |  | Lw0.1% | 32236.800^*^ | 2359.2174 | .000 | 27556.180 | 36917.420 |
|  |  | Lw0.5% | 39454.467^*^ | 2359.2174 | .000 | 34773.847 | 44135.087 |
|  |  | Lw1% | 35116.467^*^ | 2359.2174 | .000 | 30435.847 | 39797.087 |
|  |  | L50ml | 10910.200^*^ | 2359.2174 | .000 | 6229.580 | 15590.820 |
|  |  | L75ml | 8508.067^*^ | 2359.2174 | .000 | 3827.447 | 13188.687 |
|  | L50ml | control | 22379.267^*^ | 2359.2174 | .000 | 17698.647 | 27059.887 |
|  |  | C5g | 23687.733^*^ | 2359.2174 | .000 | 19007.113 | 28368.353 |
|  |  | C8g | 43509.667^*^ | 2359.2174 | .000 | 38829.047 | 48190.287 |
|  |  | C10g | 40118.200^*^ | 2359.2174 | .000 | 35437.580 | 44798.820 |
|  |  | Lw0.1% | 21326.600^*^ | 2359.2174 | .000 | 16645.980 | 26007.220 |
|  |  | Lw0.5% | 28544.267^*^ | 2359.2174 | .000 | 23863.647 | 33224.887 |
|  |  | Lw1% | 24206.267^*^ | 2359.2174 | .000 | 19525.647 | 28886.887 |
|  |  | L25ml | -10910.200^*^ | 2359.2174 | .000 | -15590.820 | -6229.580 |
|  |  | L75ml | -2402.133 | 2359.2174 | .311 | -7082.753 | 2278.487 |
|  | L75ml | control | 24781.400^*^ | 2359.2174 | .000 | 20100.780 | 29462.020 |
|  |  | C5g | 26089.867^*^ | 2359.2174 | .000 | 21409.247 | 30770.487 |
|  |  | C8g | 45911.800^*^ | 2359.2174 | .000 | 41231.180 | 50592.420 |
|  |  | C10g | 42520.333^*^ | 2359.2174 | .000 | 37839.713 | 47200.953 |
|  |  | Lw0.1% | 23728.733^*^ | 2359.2174 | .000 | 19048.113 | 28409.353 |
|  |  | Lw0.5% | 30946.400^*^ | 2359.2174 | .000 | 26265.780 | 35627.020 |
|  |  | Lw1% | 26608.400^*^ | 2359.2174 | .000 | 21927.780 | 31289.020 |
|  |  | L25ml | -8508.067^*^ | 2359.2174 | .000 | -13188.687 | -3827.447 |
|  |  | L50ml | 2402.133 | 2359.2174 | .311 | -2278.487 | 7082.753 |
| COD | control | C5g | 10405.800 | 7907.8469 | .191 | -5283.143 | 26094.743 |
|  |  | C8g | 70149.000^*^ | 7907.8469 | .000 | 54460.057 | 85837.943 |
|  |  | C10g | 60963.867^*^ | 7907.8469 | .000 | 45274.924 | 76652.810 |
|  |  | Lw0.1% | -4481.600 | 7907.8469 | .572 | -20170.543 | 11207.343 |
|  |  | Lw0.5% | 20550.000^*^ | 7907.8469 | .011 | 4861.057 | 36238.943 |
|  |  | Lw1% | 12337.800 | 7907.8469 | .122 | -3351.143 | 28026.743 |
|  |  | L25ml | -97740.933^*^ | 7907.8469 | .000 | -113429.876 | -82051.990 |
|  |  | L50ml | -73999.400^*^ | 7907.8469 | .000 | -89688.343 | -58310.457 |
|  |  | L75ml | -88474.533^*^ | 7907.8469 | .000 | -104163.476 | -72785.590 |
|  | C5g | control | -10405.800 | 7907.8469 | .191 | -26094.743 | 5283.143 |
|  |  | C8g | 59743.200^*^ | 7907.8469 | .000 | 44054.257 | 75432.143 |
|  |  | C10g | 50558.067^*^ | 7907.8469 | .000 | 34869.124 | 66247.010 |
|  |  | Lw0.1% | -14887.400 | 7907.8469 | .063 | -30576.343 | 801.543 |
|  |  | Lw0.5% | 10144.200 | 7907.8469 | .203 | -5544.743 | 25833.143 |
|  |  | Lw1% | 1932.000 | 7907.8469 | .807 | -13756.943 | 17620.943 |
|  |  | L25ml | -108146.733^*^ | 7907.8469 | .000 | -123835.676 | -92457.790 |
|  |  | L50ml | -84405.200^*^ | 7907.8469 | .000 | -100094.143 | -68716.257 |
|  |  | L75ml | -98880.333^*^ | 7907.8469 | .000 | -114569.276 | -83191.390 |
|  | C8g | control | -70149.000^*^ | 7907.8469 | .000 | -85837.943 | -54460.057 |
|  |  | C5g | -59743.200^*^ | 7907.8469 | .000 | -75432.143 | -44054.257 |
|  |  | C10g | -9185.133 | 7907.8469 | .248 | -24874.076 | 6503.810 |
|  |  | Lw0.1% | -74630.600^*^ | 7907.8469 | .000 | -90319.543 | -58941.657 |
|  |  | Lw0.5% | -49599.000^*^ | 7907.8469 | .000 | -65287.943 | -33910.057 |
|  |  | Lw1% | -57811.200^*^ | 7907.8469 | .000 | -73500.143 | -42122.257 |
|  |  | L25ml | -167889.933^*^ | 7907.8469 | .000 | -183578.876 | -152200.990 |
|  |  | L50ml | -144148.400^*^ | 7907.8469 | .000 | -159837.343 | -128459.457 |
|  |  | L75ml | -158623.533^*^ | 7907.8469 | .000 | -174312.476 | -142934.590 |
|  | C10g | control | -60963.867^*^ | 7907.8469 | .000 | -76652.810 | -45274.924 |
|  |  | C5g | -50558.067^*^ | 7907.8469 | .000 | -66247.010 | -34869.124 |
|  |  | C8g | 9185.133 | 7907.8469 | .248 | -6503.810 | 24874.076 |
|  |  | Lw0.1% | -65445.467^*^ | 7907.8469 | .000 | -81134.410 | -49756.524 |
|  |  | Lw0.5% | -40413.867^*^ | 7907.8469 | .000 | -56102.810 | -24724.924 |
|  |  | Lw1% | -48626.067^*^ | 7907.8469 | .000 | -64315.010 | -32937.124 |
|  |  | L25ml | -158704.800^*^ | 7907.8469 | .000 | -174393.743 | -143015.857 |
|  |  | L50ml | -134963.267^*^ | 7907.8469 | .000 | -150652.210 | -119274.324 |
|  |  | L75ml | -149438.400^*^ | 7907.8469 | .000 | -165127.343 | -133749.457 |
|  | Lw0.1% | control | 4481.600 | 7907.8469 | .572 | -11207.343 | 20170.543 |
|  |  | C5g | 14887.400 | 7907.8469 | .063 | -801.543 | 30576.343 |
|  |  | C8g | 74630.600^*^ | 7907.8469 | .000 | 58941.657 | 90319.543 |
|  |  | C10g | 65445.467^*^ | 7907.8469 | .000 | 49756.524 | 81134.410 |
|  |  | Lw0.5% | 25031.600^*^ | 7907.8469 | .002 | 9342.657 | 40720.543 |
|  |  | Lw1% | 16819.400^*^ | 7907.8469 | .036 | 1130.457 | 32508.343 |
|  |  | L25ml | -93259.333^*^ | 7907.8469 | .000 | -108948.276 | -77570.390 |
|  |  | L50ml | -69517.800^*^ | 7907.8469 | .000 | -85206.743 | -53828.857 |
|  |  | L75ml | -83992.933^*^ | 7907.8469 | .000 | -99681.876 | -68303.990 |
|  | Lw0.5% | control | -20550.000^*^ | 7907.8469 | .011 | -36238.943 | -4861.057 |
|  |  | C5g | -10144.200 | 7907.8469 | .203 | -25833.143 | 5544.743 |
|  |  | C8g | 49599.000^*^ | 7907.8469 | .000 | 33910.057 | 65287.943 |
|  |  | C10g | 40413.867^*^ | 7907.8469 | .000 | 24724.924 | 56102.810 |
|  |  | Lw0.1% | -25031.600^*^ | 7907.8469 | .002 | -40720.543 | -9342.657 |
|  |  | Lw1% | -8212.200 | 7907.8469 | .302 | -23901.143 | 7476.743 |
|  |  | L25ml | -118290.933^*^ | 7907.8469 | .000 | -133979.876 | -102601.990 |
|  |  | L50ml | -94549.400^*^ | 7907.8469 | .000 | -110238.343 | -78860.457 |
|  |  | L75ml | -109024.533^*^ | 7907.8469 | .000 | -124713.476 | -93335.590 |
|  | Lw1% | control | -12337.800 | 7907.8469 | .122 | -28026.743 | 3351.143 |
|  |  | C5g | -1932.000 | 7907.8469 | .807 | -17620.943 | 13756.943 |
|  |  | C8g | 57811.200^*^ | 7907.8469 | .000 | 42122.257 | 73500.143 |
|  |  | C10g | 48626.067^*^ | 7907.8469 | .000 | 32937.124 | 64315.010 |
|  |  | Lw0.1% | -16819.400^*^ | 7907.8469 | .036 | -32508.343 | -1130.457 |
|  |  | Lw0.5% | 8212.200 | 7907.8469 | .302 | -7476.743 | 23901.143 |
|  |  | L25ml | -110078.733^*^ | 7907.8469 | .000 | -125767.676 | -94389.790 |
|  |  | L50ml | -86337.200^*^ | 7907.8469 | .000 | -102026.143 | -70648.257 |
|  |  | L75ml | -100812.333^*^ | 7907.8469 | .000 | -116501.276 | -85123.390 |
|  | L25ml | control | 97740.933^*^ | 7907.8469 | .000 | 82051.990 | 113429.876 |
|  |  | C5g | 108146.733^*^ | 7907.8469 | .000 | 92457.790 | 123835.676 |
|  |  | C8g | 167889.933^*^ | 7907.8469 | .000 | 152200.990 | 183578.876 |
|  |  | C10g | 158704.800^*^ | 7907.8469 | .000 | 143015.857 | 174393.743 |
|  |  | Lw0.1% | 93259.333^*^ | 7907.8469 | .000 | 77570.390 | 108948.276 |
|  |  | Lw0.5% | 118290.933^*^ | 7907.8469 | .000 | 102601.990 | 133979.876 |
|  |  | Lw1% | 110078.733^*^ | 7907.8469 | .000 | 94389.790 | 125767.676 |
|  |  | L50ml | 23741.533^*^ | 7907.8469 | .003 | 8052.590 | 39430.476 |
|  |  | L75ml | 9266.400 | 7907.8469 | .244 | -6422.543 | 24955.343 |
|  | L50ml | control | 73999.400^*^ | 7907.8469 | .000 | 58310.457 | 89688.343 |
|  |  | C5g | 84405.200^*^ | 7907.8469 | .000 | 68716.257 | 100094.143 |
|  |  | C8g | 144148.400^*^ | 7907.8469 | .000 | 128459.457 | 159837.343 |
|  |  | C10g | 134963.267^*^ | 7907.8469 | .000 | 119274.324 | 150652.210 |
|  |  | Lw0.1% | 69517.800^*^ | 7907.8469 | .000 | 53828.857 | 85206.743 |
|  |  | Lw0.5% | 94549.400^*^ | 7907.8469 | .000 | 78860.457 | 110238.343 |
|  |  | Lw1% | 86337.200^*^ | 7907.8469 | .000 | 70648.257 | 102026.143 |
|  |  | L25ml | -23741.533^*^ | 7907.8469 | .003 | -39430.476 | -8052.590 |
|  |  | L75ml | -14475.133 | 7907.8469 | .070 | -30164.076 | 1213.810 |
|  | L75ml | control | 88474.533^*^ | 7907.8469 | .000 | 72785.590 | 104163.476 |
|  |  | C5g | 98880.333^*^ | 7907.8469 | .000 | 83191.390 | 114569.276 |
|  |  | C8g | 158623.533^*^ | 7907.8469 | .000 | 142934.590 | 174312.476 |
|  |  | C10g | 149438.400^*^ | 7907.8469 | .000 | 133749.457 | 165127.343 |
|  |  | Lw0.1% | 83992.933^*^ | 7907.8469 | .000 | 68303.990 | 99681.876 |
|  |  | Lw0.5% | 109024.533^*^ | 7907.8469 | .000 | 93335.590 | 124713.476 |
|  |  | Lw1% | 100812.333^*^ | 7907.8469 | .000 | 85123.390 | 116501.276 |
|  |  | L25ml | -9266.400 | 7907.8469 | .244 | -24955.343 | 6422.543 |
|  |  | L50ml | 14475.133 | 7907.8469 | .070 | -1213.810 | 30164.076 |
| moisture content | control | C5g | 8.029^*^ | .7351 | .000 | 6.570 | 9.487 |
|  |  | C8g | 9.067^*^ | .7351 | .000 | 7.608 | 10.525 |
|  |  | C10g | 7.391^*^ | .7351 | .000 | 5.932 | 8.849 |
|  |  | Lw0.1% | -10.243^*^ | .7351 | .000 | -11.701 | -8.784 |
|  |  | Lw0.5% | -9.969^*^ | .7351 | .000 | -11.427 | -8.510 |
|  |  | Lw1% | -8.649^*^ | .7351 | .000 | -10.107 | -7.190 |
|  |  | L25ml | -9.421^*^ | .7351 | .000 | -10.880 | -7.963 |
|  |  | L50ml | -10.279^*^ | .7351 | .000 | -11.738 | -8.821 |
|  |  | L75ml | -10.142^*^ | .7351 | .000 | -11.600 | -8.684 |
|  | C5g | control | -8.029^*^ | .7351 | .000 | -9.487 | -6.570 |
|  |  | C8g | 1.038 | .7351 | .161 | -.420 | 2.496 |
|  |  | C10g | -.638 | .7351 | .387 | -2.096 | .820 |
|  |  | Lw0.1% | -18.271^*^ | .7351 | .000 | -19.730 | -16.813 |
|  |  | Lw0.5% | -17.997^*^ | .7351 | .000 | -19.456 | -16.539 |
|  |  | Lw1% | -16.677^*^ | .7351 | .000 | -18.136 | -15.219 |
|  |  | L25ml | -17.450^*^ | .7351 | .000 | -18.908 | -15.992 |
|  |  | L50ml | -18.308^*^ | .7351 | .000 | -19.766 | -16.850 |
|  |  | L75ml | -18.171^*^ | .7351 | .000 | -19.629 | -16.712 |
|  | C8g | control | -9.067^*^ | .7351 | .000 | -10.525 | -7.608 |
|  |  | C5g | -1.038 | .7351 | .161 | -2.496 | .420 |
|  |  | C10g | -1.676^*^ | .7351 | .025 | -3.134 | -.218 |
|  |  | Lw0.1% | -19.309^*^ | .7351 | .000 | -20.768 | -17.851 |
|  |  | Lw0.5% | -19.035^*^ | .7351 | .000 | -20.494 | -17.577 |
|  |  | Lw1% | -17.715^*^ | .7351 | .000 | -19.174 | -16.257 |
|  |  | L25ml | -18.488^*^ | .7351 | .000 | -19.946 | -17.030 |
|  |  | L50ml | -19.346^*^ | .7351 | .000 | -20.804 | -17.888 |
|  |  | L75ml | -19.209^*^ | .7351 | .000 | -20.667 | -17.750 |
|  | C10g | control | -7.391^*^ | .7351 | .000 | -8.849 | -5.932 |
|  |  | C5g | .638 | .7351 | .387 | -.820 | 2.096 |
|  |  | C8g | 1.676^*^ | .7351 | .025 | .218 | 3.134 |
|  |  | Lw0.1% | -17.633^*^ | .7351 | .000 | -19.092 | -16.175 |
|  |  | Lw0.5% | -17.359^*^ | .7351 | .000 | -18.818 | -15.901 |
|  |  | Lw1% | -16.039^*^ | .7351 | .000 | -17.498 | -14.581 |
|  |  | L25ml | -16.812^*^ | .7351 | .000 | -18.270 | -15.354 |
|  |  | L50ml | -17.670^*^ | .7351 | .000 | -19.128 | -16.212 |
|  |  | L75ml | -17.533^*^ | .7351 | .000 | -18.991 | -16.074 |
|  | Lw0.1% | control | 10.243^*^ | .7351 | .000 | 8.784 | 11.701 |
|  |  | C5g | 18.271^*^ | .7351 | .000 | 16.813 | 19.730 |
|  |  | C8g | 19.309^*^ | .7351 | .000 | 17.851 | 20.768 |
|  |  | C10g | 17.633^*^ | .7351 | .000 | 16.175 | 19.092 |
|  |  | Lw0.5% | .274 | .7351 | .710 | -1.184 | 1.732 |
|  |  | Lw1% | 1.594^*^ | .7351 | .032 | .136 | 3.052 |
|  |  | L25ml | .821 | .7351 | .267 | -.637 | 2.280 |
|  |  | L50ml | -.037 | .7351 | .960 | -1.495 | 1.422 |
|  |  | L75ml | .101 | .7351 | .891 | -1.358 | 1.559 |
|  | Lw0.5% | control | 9.969^*^ | .7351 | .000 | 8.510 | 11.427 |
|  |  | C5g | 17.997^*^ | .7351 | .000 | 16.539 | 19.456 |
|  |  | C8g | 19.035^*^ | .7351 | .000 | 17.577 | 20.494 |
|  |  | C10g | 17.359^*^ | .7351 | .000 | 15.901 | 18.818 |
|  |  | Lw0.1% | -.274 | .7351 | .710 | -1.732 | 1.184 |
|  |  | Lw1% | 1.320 | .7351 | .076 | -.138 | 2.778 |
|  |  | L25ml | .547 | .7351 | .458 | -.911 | 2.006 |
|  |  | L50ml | -.311 | .7351 | .673 | -1.769 | 1.148 |
|  |  | L75ml | -.173 | .7351 | .814 | -1.632 | 1.285 |
|  | Lw1% | control | 8.649^*^ | .7351 | .000 | 7.190 | 10.107 |
|  |  | C5g | 16.677^*^ | .7351 | .000 | 15.219 | 18.136 |
|  |  | C8g | 17.715^*^ | .7351 | .000 | 16.257 | 19.174 |
|  |  | C10g | 16.039^*^ | .7351 | .000 | 14.581 | 17.498 |
|  |  | Lw0.1% | -1.594^*^ | .7351 | .032 | -3.052 | -.136 |
|  |  | Lw0.5% | -1.320 | .7351 | .076 | -2.778 | .138 |
|  |  | L25ml | -.773 | .7351 | .296 | -2.231 | .686 |
|  |  | L50ml | -1.631^*^ | .7351 | .029 | -3.089 | -.172 |
|  |  | L75ml | -1.493^*^ | .7351 | .045 | -2.952 | -.035 |
|  | L25ml | control | 9.421^*^ | .7351 | .000 | 7.963 | 10.880 |
|  |  | C5g | 17.450^*^ | .7351 | .000 | 15.992 | 18.908 |
|  |  | C8g | 18.488^*^ | .7351 | .000 | 17.030 | 19.946 |
|  |  | C10g | 16.812^*^ | .7351 | .000 | 15.354 | 18.270 |
|  |  | Lw0.1% | -.821 | .7351 | .267 | -2.280 | .637 |
|  |  | Lw0.5% | -.547 | .7351 | .458 | -2.006 | .911 |
|  |  | Lw1% | .773 | .7351 | .296 | -.686 | 2.231 |
|  |  | L50ml | -.858 | .7351 | .246 | -2.316 | .600 |
|  |  | L75ml | -.721 | .7351 | .329 | -2.179 | .738 |
|  | L50ml | control | 10.279^*^ | .7351 | .000 | 8.821 | 11.738 |
|  |  | C5g | 18.308^*^ | .7351 | .000 | 16.850 | 19.766 |
|  |  | C8g | 19.346^*^ | .7351 | .000 | 17.888 | 20.804 |
|  |  | C10g | 17.670^*^ | .7351 | .000 | 16.212 | 19.128 |
|  |  | Lw0.1% | .037 | .7351 | .960 | -1.422 | 1.495 |
|  |  | Lw0.5% | .311 | .7351 | .673 | -1.148 | 1.769 |
|  |  | Lw1% | 1.631^*^ | .7351 | .029 | .172 | 3.089 |
|  |  | L25ml | .858 | .7351 | .246 | -.600 | 2.316 |
|  |  | L75ml | .137 | .7351 | .852 | -1.321 | 1.596 |
|  | L75ml | control | 10.142^*^ | .7351 | .000 | 8.684 | 11.600 |
|  |  | C5g | 18.171^*^ | .7351 | .000 | 16.712 | 19.629 |
|  |  | C8g | 19.209^*^ | .7351 | .000 | 17.750 | 20.667 |
|  |  | C10g | 17.533^*^ | .7351 | .000 | 16.074 | 18.991 |
|  |  | Lw0.1% | -.101 | .7351 | .891 | -1.559 | 1.358 |
|  |  | Lw0.5% | .173 | .7351 | .814 | -1.285 | 1.632 |
|  |  | Lw1% | 1.493^*^ | .7351 | .045 | .035 | 2.952 |
|  |  | L25ml | .721 | .7351 | .329 | -.738 | 2.179 |
|  |  | L50ml | -.137 | .7351 | .852 | -1.596 | 1.321 |
| total coliforms | control | C5g | 39.867^*^ | 1.6185 | .000 | 36.656 | 43.078 |
|  |  | C8g | 40.800^*^ | 1.6185 | .000 | 37.589 | 44.011 |
|  |  | C10g | 50.467^*^ | 1.6185 | .000 | 47.256 | 53.678 |
|  |  | Lw0.1% | 4.533^*^ | 1.6185 | .006 | 1.322 | 7.744 |
|  |  | Lw0.5% | 14.867^*^ | 1.6185 | .000 | 11.656 | 18.078 |
|  |  | Lw1% | 24.067^*^ | 1.6185 | .000 | 20.856 | 27.278 |
|  |  | L25ml | 50.533^*^ | 1.6185 | .000 | 47.322 | 53.744 |
|  |  | L50ml | 50.000^*^ | 1.6185 | .000 | 46.789 | 53.211 |
|  |  | L75ml | 51.200^*^ | 1.6185 | .000 | 47.989 | 54.411 |
|  | C5g | control | -39.867^*^ | 1.6185 | .000 | -43.078 | -36.656 |
|  |  | C8g | .933 | 1.6185 | .565 | -2.278 | 4.144 |
|  |  | C10g | 10.600^*^ | 1.6185 | .000 | 7.389 | 13.811 |
|  |  | Lw0.1% | -35.333^*^ | 1.6185 | .000 | -38.544 | -32.122 |
|  |  | Lw0.5% | -25.000^*^ | 1.6185 | .000 | -28.211 | -21.789 |
|  |  | Lw1% | -15.800^*^ | 1.6185 | .000 | -19.011 | -12.589 |
|  |  | L25ml | 10.667^*^ | 1.6185 | .000 | 7.456 | 13.878 |
|  |  | L50ml | 10.133^*^ | 1.6185 | .000 | 6.922 | 13.344 |
|  |  | L75ml | 11.333^*^ | 1.6185 | .000 | 8.122 | 14.544 |
|  | C8g | control | -40.800^*^ | 1.6185 | .000 | -44.011 | -37.589 |
|  |  | C5g | -.933 | 1.6185 | .565 | -4.144 | 2.278 |
|  |  | C10g | 9.667^*^ | 1.6185 | .000 | 6.456 | 12.878 |
|  |  | Lw0.1% | -36.267^*^ | 1.6185 | .000 | -39.478 | -33.056 |
|  |  | Lw0.5% | -25.933^*^ | 1.6185 | .000 | -29.144 | -22.722 |
|  |  | Lw1% | -16.733^*^ | 1.6185 | .000 | -19.944 | -13.522 |
|  |  | L25ml | 9.733^*^ | 1.6185 | .000 | 6.522 | 12.944 |
|  |  | L50ml | 9.200^*^ | 1.6185 | .000 | 5.989 | 12.411 |
|  |  | L75ml | 10.400^*^ | 1.6185 | .000 | 7.189 | 13.611 |
|  | C10g | control | -50.467^*^ | 1.6185 | .000 | -53.678 | -47.256 |
|  |  | C5g | -10.600^*^ | 1.6185 | .000 | -13.811 | -7.389 |
|  |  | C8g | -9.667^*^ | 1.6185 | .000 | -12.878 | -6.456 |
|  |  | Lw0.1% | -45.933^*^ | 1.6185 | .000 | -49.144 | -42.722 |
|  |  | Lw0.5% | -35.600^*^ | 1.6185 | .000 | -38.811 | -32.389 |
|  |  | Lw1% | -26.400^*^ | 1.6185 | .000 | -29.611 | -23.189 |
|  |  | L25ml | .067 | 1.6185 | .967 | -3.144 | 3.278 |
|  |  | L50ml | -.467 | 1.6185 | .774 | -3.678 | 2.744 |
|  |  | L75ml | .733 | 1.6185 | .651 | -2.478 | 3.944 |
|  | Lw0.1% | control | -4.533^*^ | 1.6185 | .006 | -7.744 | -1.322 |
|  |  | C5g | 35.333^*^ | 1.6185 | .000 | 32.122 | 38.544 |
|  |  | C8g | 36.267^*^ | 1.6185 | .000 | 33.056 | 39.478 |
|  |  | C10g | 45.933^*^ | 1.6185 | .000 | 42.722 | 49.144 |
|  |  | Lw0.5% | 10.333^*^ | 1.6185 | .000 | 7.122 | 13.544 |
|  |  | Lw1% | 19.533^*^ | 1.6185 | .000 | 16.322 | 22.744 |
|  |  | L25ml | 46.000^*^ | 1.6185 | .000 | 42.789 | 49.211 |
|  |  | L50ml | 45.467^*^ | 1.6185 | .000 | 42.256 | 48.678 |
|  |  | L75ml | 46.667^*^ | 1.6185 | .000 | 43.456 | 49.878 |
|  | Lw0.5% | control | -14.867^*^ | 1.6185 | .000 | -18.078 | -11.656 |
|  |  | C5g | 25.000^*^ | 1.6185 | .000 | 21.789 | 28.211 |
|  |  | C8g | 25.933^*^ | 1.6185 | .000 | 22.722 | 29.144 |
|  |  | C10g | 35.600^*^ | 1.6185 | .000 | 32.389 | 38.811 |
|  |  | Lw0.1% | -10.333^*^ | 1.6185 | .000 | -13.544 | -7.122 |
|  |  | Lw1% | 9.200^*^ | 1.6185 | .000 | 5.989 | 12.411 |
|  |  | L25ml | 35.667^*^ | 1.6185 | .000 | 32.456 | 38.878 |
|  |  | L50ml | 35.133^*^ | 1.6185 | .000 | 31.922 | 38.344 |
|  |  | L75ml | 36.333^*^ | 1.6185 | .000 | 33.122 | 39.544 |
|  | Lw1% | control | -24.067^*^ | 1.6185 | .000 | -27.278 | -20.856 |
|  |  | C5g | 15.800^*^ | 1.6185 | .000 | 12.589 | 19.011 |
|  |  | C8g | 16.733^*^ | 1.6185 | .000 | 13.522 | 19.944 |
|  |  | C10g | 26.400^*^ | 1.6185 | .000 | 23.189 | 29.611 |
|  |  | Lw0.1% | -19.533^*^ | 1.6185 | .000 | -22.744 | -16.322 |
|  |  | Lw0.5% | -9.200^*^ | 1.6185 | .000 | -12.411 | -5.989 |
|  |  | L25ml | 26.467^*^ | 1.6185 | .000 | 23.256 | 29.678 |
|  |  | L50ml | 25.933^*^ | 1.6185 | .000 | 22.722 | 29.144 |
|  |  | L75ml | 27.133^*^ | 1.6185 | .000 | 23.922 | 30.344 |
|  | L25ml | control | -50.533^*^ | 1.6185 | .000 | -53.744 | -47.322 |
|  |  | C5g | -10.667^*^ | 1.6185 | .000 | -13.878 | -7.456 |
|  |  | C8g | -9.733^*^ | 1.6185 | .000 | -12.944 | -6.522 |
|  |  | C10g | -.067 | 1.6185 | .967 | -3.278 | 3.144 |
|  |  | Lw0.1% | -46.000^*^ | 1.6185 | .000 | -49.211 | -42.789 |
|  |  | Lw0.5% | -35.667^*^ | 1.6185 | .000 | -38.878 | -32.456 |
|  |  | Lw1% | -26.467^*^ | 1.6185 | .000 | -29.678 | -23.256 |
|  |  | L50ml | -.533 | 1.6185 | .742 | -3.744 | 2.678 |
|  |  | L75ml | .667 | 1.6185 | .681 | -2.544 | 3.878 |
|  | L50ml | control | -50.000^*^ | 1.6185 | .000 | -53.211 | -46.789 |
|  |  | C5g | -10.133^*^ | 1.6185 | .000 | -13.344 | -6.922 |
|  |  | C8g | -9.200^*^ | 1.6185 | .000 | -12.411 | -5.989 |
|  |  | C10g | .467 | 1.6185 | .774 | -2.744 | 3.678 |
|  |  | Lw0.1% | -45.467^*^ | 1.6185 | .000 | -48.678 | -42.256 |
|  |  | Lw0.5% | -35.133^*^ | 1.6185 | .000 | -38.344 | -31.922 |
|  |  | Lw1% | -25.933^*^ | 1.6185 | .000 | -29.144 | -22.722 |
|  |  | L25ml | .533 | 1.6185 | .742 | -2.678 | 3.744 |
|  |  | L75ml | 1.200 | 1.6185 | .460 | -2.011 | 4.411 |
|  | L75ml | control | -51.200^*^ | 1.6185 | .000 | -54.411 | -47.989 |
|  |  | C5g | -11.333^*^ | 1.6185 | .000 | -14.544 | -8.122 |
|  |  | C8g | -10.400^*^ | 1.6185 | .000 | -13.611 | -7.189 |
|  |  | C10g | -.733 | 1.6185 | .651 | -3.944 | 2.478 |
|  |  | Lw0.1% | -46.667^*^ | 1.6185 | .000 | -49.878 | -43.456 |
|  |  | Lw0.5% | -36.333^*^ | 1.6185 | .000 | -39.544 | -33.122 |
|  |  | Lw1% | -27.133^*^ | 1.6185 | .000 | -30.344 | -23.922 |
|  |  | L25ml | -.667 | 1.6185 | .681 | -3.878 | 2.544 |
|  |  | L50ml | -1.200 | 1.6185 | .460 | -4.411 | 2.011 |
| helminth eggs | control | C5g | -11.400 | 19.0175 | .550 | -49.130 | 26.330 |
|  |  | C8g | -43.067^*^ | 19.0175 | .026 | -80.797 | -5.337 |
|  |  | C10g | -27.200 | 19.0175 | .156 | -64.930 | 10.530 |
|  |  | Lw0.1% | 34.733 | 19.0175 | .071 | -2.997 | 72.463 |
|  |  | Lw0.5% | 48.933^*^ | 19.0175 | .012 | 11.203 | 86.663 |
|  |  | Lw1% | -114.333^*^ | 19.0175 | .000 | -152.063 | -76.603 |
|  |  | L25ml | -3.133 | 19.0175 | .869 | -40.863 | 34.597 |
|  |  | L50ml | 61.800^*^ | 19.0175 | .002 | 24.070 | 99.530 |
|  |  | L75ml | 40.600^*^ | 19.0175 | .035 | 2.870 | 78.330 |
|  | C5g | control | 11.400 | 19.0175 | .550 | -26.330 | 49.130 |
|  |  | C8g | -31.667 | 19.0175 | .099 | -69.397 | 6.063 |
|  |  | C10g | -15.800 | 19.0175 | .408 | -53.530 | 21.930 |
|  |  | Lw0.1% | 46.133^*^ | 19.0175 | .017 | 8.403 | 83.863 |
|  |  | Lw0.5% | 60.333^*^ | 19.0175 | .002 | 22.603 | 98.063 |
|  |  | Lw1% | -102.933^*^ | 19.0175 | .000 | -140.663 | -65.203 |
|  |  | L25ml | 8.267 | 19.0175 | .665 | -29.463 | 45.997 |
|  |  | L50ml | 73.200^*^ | 19.0175 | .000 | 35.470 | 110.930 |
|  |  | L75ml | 52.000^*^ | 19.0175 | .007 | 14.270 | 89.730 |
|  | C8g | control | 43.067^*^ | 19.0175 | .026 | 5.337 | 80.797 |
|  |  | C5g | 31.667 | 19.0175 | .099 | -6.063 | 69.397 |
|  |  | C10g | 15.867 | 19.0175 | .406 | -21.863 | 53.597 |
|  |  | Lw0.1% | 77.800^*^ | 19.0175 | .000 | 40.070 | 115.530 |
|  |  | Lw0.5% | 92.000^*^ | 19.0175 | .000 | 54.270 | 129.730 |
|  |  | Lw1% | -71.267^*^ | 19.0175 | .000 | -108.997 | -33.537 |
|  |  | L25ml | 39.933^*^ | 19.0175 | .038 | 2.203 | 77.663 |
|  |  | L50ml | 104.867^*^ | 19.0175 | .000 | 67.137 | 142.597 |
|  |  | L75ml | 83.667^*^ | 19.0175 | .000 | 45.937 | 121.397 |
|  | C10g | control | 27.200 | 19.0175 | .156 | -10.530 | 64.930 |
|  |  | C5g | 15.800 | 19.0175 | .408 | -21.930 | 53.530 |
|  |  | C8g | -15.867 | 19.0175 | .406 | -53.597 | 21.863 |
|  |  | Lw0.1% | 61.933^*^ | 19.0175 | .002 | 24.203 | 99.663 |
|  |  | Lw0.5% | 76.133^*^ | 19.0175 | .000 | 38.403 | 113.863 |
|  |  | Lw1% | -87.133^*^ | 19.0175 | .000 | -124.863 | -49.403 |
|  |  | L25ml | 24.067 | 19.0175 | .209 | -13.663 | 61.797 |
|  |  | L50ml | 89.000^*^ | 19.0175 | .000 | 51.270 | 126.730 |
|  |  | L75ml | 67.800^*^ | 19.0175 | .001 | 30.070 | 105.530 |
|  | Lw0.1% | control | -34.733 | 19.0175 | .071 | -72.463 | 2.997 |
|  |  | C5g | -46.133^*^ | 19.0175 | .017 | -83.863 | -8.403 |
|  |  | C8g | -77.800^*^ | 19.0175 | .000 | -115.530 | -40.070 |
|  |  | C10g | -61.933^*^ | 19.0175 | .002 | -99.663 | -24.203 |
|  |  | Lw0.5% | 14.200 | 19.0175 | .457 | -23.530 | 51.930 |
|  |  | Lw1% | -149.067^*^ | 19.0175 | .000 | -186.797 | -111.337 |
|  |  | L25ml | -37.867^*^ | 19.0175 | .049 | -75.597 | -.137 |
|  |  | L50ml | 27.067 | 19.0175 | .158 | -10.663 | 64.797 |
|  |  | L75ml | 5.867 | 19.0175 | .758 | -31.863 | 43.597 |
|  | Lw0.5% | control | -48.933^*^ | 19.0175 | .012 | -86.663 | -11.203 |
|  |  | C5g | -60.333^*^ | 19.0175 | .002 | -98.063 | -22.603 |
|  |  | C8g | -92.000^*^ | 19.0175 | .000 | -129.730 | -54.270 |
|  |  | C10g | -76.133^*^ | 19.0175 | .000 | -113.863 | -38.403 |
|  |  | Lw0.1% | -14.200 | 19.0175 | .457 | -51.930 | 23.530 |
|  |  | Lw1% | -163.267^*^ | 19.0175 | .000 | -200.997 | -125.537 |
|  |  | L25ml | -52.067^*^ | 19.0175 | .007 | -89.797 | -14.337 |
|  |  | L50ml | 12.867 | 19.0175 | .500 | -24.863 | 50.597 |
|  |  | L75ml | -8.333 | 19.0175 | .662 | -46.063 | 29.397 |
|  | Lw1% | control | 114.333^*^ | 19.0175 | .000 | 76.603 | 152.063 |
|  |  | C5g | 102.933^*^ | 19.0175 | .000 | 65.203 | 140.663 |
|  |  | C8g | 71.267^*^ | 19.0175 | .000 | 33.537 | 108.997 |
|  |  | C10g | 87.133^*^ | 19.0175 | .000 | 49.403 | 124.863 |
|  |  | Lw0.1% | 149.067^*^ | 19.0175 | .000 | 111.337 | 186.797 |
|  |  | Lw0.5% | 163.267^*^ | 19.0175 | .000 | 125.537 | 200.997 |
|  |  | L25ml | 111.200^*^ | 19.0175 | .000 | 73.470 | 148.930 |
|  |  | L50ml | 176.133^*^ | 19.0175 | .000 | 138.403 | 213.863 |
|  |  | L75ml | 154.933^*^ | 19.0175 | .000 | 117.203 | 192.663 |
|  | L25ml | control | 3.133 | 19.0175 | .869 | -34.597 | 40.863 |
|  |  | C5g | -8.267 | 19.0175 | .665 | -45.997 | 29.463 |
|  |  | C8g | -39.933^*^ | 19.0175 | .038 | -77.663 | -2.203 |
|  |  | C10g | -24.067 | 19.0175 | .209 | -61.797 | 13.663 |
|  |  | Lw0.1% | 37.867^*^ | 19.0175 | .049 | .137 | 75.597 |
|  |  | Lw0.5% | 52.067^*^ | 19.0175 | .007 | 14.337 | 89.797 |
|  |  | Lw1% | -111.200^*^ | 19.0175 | .000 | -148.930 | -73.470 |
|  |  | L50ml | 64.933^*^ | 19.0175 | .001 | 27.203 | 102.663 |
|  |  | L75ml | 43.733^*^ | 19.0175 | .024 | 6.003 | 81.463 |
|  | L50ml | control | -61.800^*^ | 19.0175 | .002 | -99.530 | -24.070 |
|  |  | C5g | -73.200^*^ | 19.0175 | .000 | -110.930 | -35.470 |
|  |  | C8g | -104.867^*^ | 19.0175 | .000 | -142.597 | -67.137 |
|  |  | C10g | -89.000^*^ | 19.0175 | .000 | -126.730 | -51.270 |
|  |  | Lw0.1% | -27.067 | 19.0175 | .158 | -64.797 | 10.663 |
|  |  | Lw0.5% | -12.867 | 19.0175 | .500 | -50.597 | 24.863 |
|  |  | Lw1% | -176.133^*^ | 19.0175 | .000 | -213.863 | -138.403 |
|  |  | L25ml | -64.933^*^ | 19.0175 | .001 | -102.663 | -27.203 |
|  |  | L75ml | -21.200 | 19.0175 | .268 | -58.930 | 16.530 |
|  | L75ml | control | -40.600^*^ | 19.0175 | .035 | -78.330 | -2.870 |
|  |  | C5g | -52.000^*^ | 19.0175 | .007 | -89.730 | -14.270 |
|  |  | C8g | -83.667^*^ | 19.0175 | .000 | -121.397 | -45.937 |
|  |  | C10g | -67.800^*^ | 19.0175 | .001 | -105.530 | -30.070 |
|  |  | Lw0.1% | -5.867 | 19.0175 | .758 | -43.597 | 31.863 |
|  |  | Lw0.5% | 8.333 | 19.0175 | .662 | -29.397 | 46.063 |
|  |  | Lw1% | -154.933^*^ | 19.0175 | .000 | -192.663 | -117.203 |
|  |  | L25ml | -43.733^*^ | 19.0175 | .024 | -81.463 | -6.003 |
|  |  | L50ml | 21.200 | 19.0175 | .268 | -16.530 | 58.930 |
| Based on observed means.  The error term is Mean Square(Error) = 2712.487. | | | | | | | |
| *. The mean difference is significant at 0.05 | | | | | | | |
