# Supplementary material for: Toilet chemical additives and their effect on faecal sludge characteristics
Source: Heliyon. 2020 Sep 23;6(9):e04998. doi: 10.1016/j.heliyon.2020.e04998 (PMC7511817; doi:10.1016/j.heliyon.2020.e04998)
Supplement: Supplementary Table A4.docx [file mmc4.docx]

| **Table A4. Multiple Comparisons** | | | | | | | |
| --- | --- | --- | --- | --- | --- | --- | --- |
| LSD |  |  |  |  |  |  |  |
| Dependent Variable | | | Mean Difference (I-J) | Std. Error | Sig. | 95% Confidence Interval | |
|  |  |  |  |  |  | Lower Bound | Upper Bound |
| BOD5 | wk0 | wk1 | 12344.267^*^ | 1668.2186 | .000 | 9034.568 | 15653.965 |
|  |  | wk2 | 17375.900^*^ | 1668.2186 | .000 | 14066.202 | 20685.598 |
|  |  | wk3 | 20181.033^*^ | 1668.2186 | .000 | 16871.335 | 23490.732 |
|  |  | wk4 | 22010.633^*^ | 1668.2186 | .000 | 18700.935 | 25320.332 |
|  | wk1 | wk0 | -12344.267^*^ | 1668.2186 | .000 | -15653.965 | -9034.568 |
|  |  | wk2 | 5031.633^*^ | 1668.2186 | .003 | 1721.935 | 8341.332 |
|  |  | wk3 | 7836.767^*^ | 1668.2186 | .000 | 4527.068 | 11146.465 |
|  |  | wk4 | 9666.367^*^ | 1668.2186 | .000 | 6356.668 | 12976.065 |
|  | wk2 | wk0 | -17375.900^*^ | 1668.2186 | .000 | -20685.598 | -14066.202 |
|  |  | wk1 | -5031.633^*^ | 1668.2186 | .003 | -8341.332 | -1721.935 |
|  |  | wk3 | 2805.133 | 1668.2186 | .096 | -504.565 | 6114.832 |
|  |  | wk4 | 4634.733^*^ | 1668.2186 | .007 | 1325.035 | 7944.432 |
|  | wk3 | wk0 | -20181.033^*^ | 1668.2186 | .000 | -23490.732 | -16871.335 |
|  |  | wk1 | -7836.767^*^ | 1668.2186 | .000 | -11146.465 | -4527.068 |
|  |  | wk2 | -2805.133 | 1668.2186 | .096 | -6114.832 | 504.565 |
|  |  | wk4 | 1829.600 | 1668.2186 | .275 | -1480.098 | 5139.298 |
|  | wk4 | wk0 | -22010.633^*^ | 1668.2186 | .000 | -25320.332 | -18700.935 |
|  |  | wk1 | -9666.367^*^ | 1668.2186 | .000 | -12976.065 | -6356.668 |
|  |  | wk2 | -4634.733^*^ | 1668.2186 | .007 | -7944.432 | -1325.035 |
|  |  | wk3 | -1829.600 | 1668.2186 | .275 | -5139.298 | 1480.098 |
| COD | wk0 | wk1 | 49393.033^*^ | 5591.6921 | .000 | 38299.275 | 60486.791 |
|  |  | wk2 | 69609.133^*^ | 5591.6921 | .000 | 58515.375 | 80702.891 |
|  |  | wk3 | 83703.933^*^ | 5591.6921 | .000 | 72610.175 | 94797.691 |
|  |  | wk4 | 95200.233^*^ | 5591.6921 | .000 | 84106.475 | 106293.991 |
|  | wk1 | wk0 | -49393.033^*^ | 5591.6921 | .000 | -60486.791 | -38299.275 |
|  |  | wk2 | 20216.100^*^ | 5591.6921 | .000 | 9122.342 | 31309.858 |
|  |  | wk3 | 34310.900^*^ | 5591.6921 | .000 | 23217.142 | 45404.658 |
|  |  | wk4 | 45807.200^*^ | 5591.6921 | .000 | 34713.442 | 56900.958 |
|  | wk2 | wk0 | -69609.133^*^ | 5591.6921 | .000 | -80702.891 | -58515.375 |
|  |  | wk1 | -20216.100^*^ | 5591.6921 | .000 | -31309.858 | -9122.342 |
|  |  | wk3 | 14094.800^*^ | 5591.6921 | .013 | 3001.042 | 25188.558 |
|  |  | wk4 | 25591.100^*^ | 5591.6921 | .000 | 14497.342 | 36684.858 |
|  | wk3 | wk0 | -83703.933^*^ | 5591.6921 | .000 | -94797.691 | -72610.175 |
|  |  | wk1 | -34310.900^*^ | 5591.6921 | .000 | -45404.658 | -23217.142 |
|  |  | wk2 | -14094.800^*^ | 5591.6921 | .013 | -25188.558 | -3001.042 |
|  |  | wk4 | 11496.300^*^ | 5591.6921 | .042 | 402.542 | 22590.058 |
|  | wk4 | wk0 | -95200.233^*^ | 5591.6921 | .000 | -106293.991 | -84106.475 |
|  |  | wk1 | -45807.200^*^ | 5591.6921 | .000 | -56900.958 | -34713.442 |
|  |  | wk2 | -25591.100^*^ | 5591.6921 | .000 | -36684.858 | -14497.342 |
|  |  | wk3 | -11496.300^*^ | 5591.6921 | .042 | -22590.058 | -402.542 |
| moisture content | wk0 | wk1 | 5.295^*^ | .5198 | .000 | 4.264 | 6.326 |
|  |  | wk2 | 14.761^*^ | .5198 | .000 | 13.730 | 15.792 |
|  |  | wk3 | 16.634^*^ | .5198 | .000 | 15.602 | 17.665 |
|  |  | wk4 | 22.337^*^ | .5198 | .000 | 21.306 | 23.368 |
|  | wk1 | wk0 | -5.295^*^ | .5198 | .000 | -6.326 | -4.264 |
|  |  | wk2 | 9.466^*^ | .5198 | .000 | 8.435 | 10.497 |
|  |  | wk3 | 11.339^*^ | .5198 | .000 | 10.307 | 12.370 |
|  |  | wk4 | 17.042^*^ | .5198 | .000 | 16.011 | 18.073 |
|  | wk2 | wk0 | -14.761^*^ | .5198 | .000 | -15.792 | -13.730 |
|  |  | wk1 | -9.466^*^ | .5198 | .000 | -10.497 | -8.435 |
|  |  | wk3 | 1.873^*^ | .5198 | .000 | .841 | 2.904 |
|  |  | wk4 | 7.576^*^ | .5198 | .000 | 6.545 | 8.607 |
|  | wk3 | wk0 | -16.634^*^ | .5198 | .000 | -17.665 | -15.602 |
|  |  | wk1 | -11.339^*^ | .5198 | .000 | -12.370 | -10.307 |
|  |  | wk2 | -1.873^*^ | .5198 | .000 | -2.904 | -.841 |
|  |  | wk4 | 5.703^*^ | .5198 | .000 | 4.672 | 6.735 |
|  | wk4 | wk0 | -22.337^*^ | .5198 | .000 | -23.368 | -21.306 |
|  |  | wk1 | -17.042^*^ | .5198 | .000 | -18.073 | -16.011 |
|  |  | wk2 | -7.576^*^ | .5198 | .000 | -8.607 | -6.545 |
|  |  | wk3 | -5.703^*^ | .5198 | .000 | -6.735 | -4.672 |
| total coliforms | wk0 | wk1 | 14.233^*^ | 1.1445 | .000 | 11.963 | 16.504 |
|  |  | wk2 | 30.933^*^ | 1.1445 | .000 | 28.663 | 33.204 |
|  |  | wk3 | 40.400^*^ | 1.1445 | .000 | 38.129 | 42.671 |
|  |  | wk4 | 44.933^*^ | 1.1445 | .000 | 42.663 | 47.204 |
|  | wk1 | wk0 | -14.233^*^ | 1.1445 | .000 | -16.504 | -11.963 |
|  |  | wk2 | 16.700^*^ | 1.1445 | .000 | 14.429 | 18.971 |
|  |  | wk3 | 26.167^*^ | 1.1445 | .000 | 23.896 | 28.437 |
|  |  | wk4 | 30.700^*^ | 1.1445 | .000 | 28.429 | 32.971 |
|  | wk2 | wk0 | -30.933^*^ | 1.1445 | .000 | -33.204 | -28.663 |
|  |  | wk1 | -16.700^*^ | 1.1445 | .000 | -18.971 | -14.429 |
|  |  | wk3 | 9.467^*^ | 1.1445 | .000 | 7.196 | 11.737 |
|  |  | wk4 | 14.000^*^ | 1.1445 | .000 | 11.729 | 16.271 |
|  | wk3 | wk0 | -40.400^*^ | 1.1445 | .000 | -42.671 | -38.129 |
|  |  | wk1 | -26.167^*^ | 1.1445 | .000 | -28.437 | -23.896 |
|  |  | wk2 | -9.467^*^ | 1.1445 | .000 | -11.737 | -7.196 |
|  |  | wk4 | 4.533^*^ | 1.1445 | .000 | 2.263 | 6.804 |
|  | wk4 | wk0 | -44.933^*^ | 1.1445 | .000 | -47.204 | -42.663 |
|  |  | wk1 | -30.700^*^ | 1.1445 | .000 | -32.971 | -28.429 |
|  |  | wk2 | -14.000^*^ | 1.1445 | .000 | -16.271 | -11.729 |
|  |  | wk3 | -4.533^*^ | 1.1445 | .000 | -6.804 | -2.263 |
| helminth eggs | wk0 | wk1 | 224.633^*^ | 13.4474 | .000 | 197.954 | 251.313 |
|  |  | wk2 | 329.933^*^ | 13.4474 | .000 | 303.254 | 356.613 |
|  |  | wk3 | 372.167^*^ | 13.4474 | .000 | 345.487 | 398.846 |
|  |  | wk4 | 403.400^*^ | 13.4474 | .000 | 376.721 | 430.079 |
|  | wk1 | wk0 | -224.633^*^ | 13.4474 | .000 | -251.313 | -197.954 |
|  |  | wk2 | 105.300^*^ | 13.4474 | .000 | 78.621 | 131.979 |
|  |  | wk3 | 147.533^*^ | 13.4474 | .000 | 120.854 | 174.213 |
|  |  | wk4 | 178.767^*^ | 13.4474 | .000 | 152.087 | 205.446 |
|  | wk2 | wk0 | -329.933^*^ | 13.4474 | .000 | -356.613 | -303.254 |
|  |  | wk1 | -105.300^*^ | 13.4474 | .000 | -131.979 | -78.621 |
|  |  | wk3 | 42.233^*^ | 13.4474 | .002 | 15.554 | 68.913 |
|  |  | wk4 | 73.467^*^ | 13.4474 | .000 | 46.787 | 100.146 |
|  | wk3 | wk0 | -372.167^*^ | 13.4474 | .000 | -398.846 | -345.487 |
|  |  | wk1 | -147.533^*^ | 13.4474 | .000 | -174.213 | -120.854 |
|  |  | wk2 | -42.233^*^ | 13.4474 | .002 | -68.913 | -15.554 |
|  |  | wk4 | 31.233^*^ | 13.4474 | .022 | 4.554 | 57.913 |
|  | wk4 | wk0 | -403.400^*^ | 13.4474 | .000 | -430.079 | -376.721 |
|  |  | wk1 | -178.767^*^ | 13.4474 | .000 | -205.446 | -152.087 |
|  |  | wk2 | -73.467^*^ | 13.4474 | .000 | -100.146 | -46.787 |
|  |  | wk3 | -31.233^*^ | 13.4474 | .022 | -57.913 | -4.554 |
| Based on observed means.  The error term is Mean Square(Error) = 2712.487. | | | | | | | |
| *. The mean difference is significant at 0.05 | | | | | | | |
